# Differential proteomic analysis of *Clostridium perfringens *ATCC13124; identification of dominant, surface and structure associated proteins

**DOI:** 10.1186/1471-2180-9-162

**Published:** 2009-08-10

**Authors:** Syed Imteyaz Alam, Sunita Bansod, Ravi Bhushan Kumar, Nabonita Sengupta, Lokendra Singh

**Affiliations:** 1 Biotechnology Division, Defence Research & Development Establishment, Gwalior-474002, India

## Abstract

**Background:**

*Clostridium perfringens *is a medically important clostridial pathogen causing diseases in man and animals. To invade, multiply and colonize tissues of the host, a pathogen must be able to evade host immune system, and obtain nutrients essential for growth. The factors involved in these complex processes are largely unknown and of crucial importance to understanding microbial pathogenesis. Many of the virulence determinants and putative vaccine candidates for bacterial pathogens are known to be surface localized.

**Results:**

Using 2-DE mass spectrometry strategy, we identified major surface (22) and cell envelope (10) proteins from *Clostridium perfringens *ATCC13124 and those differentially expressed (11) in cells grown on cooked meat medium (CMM) in comparison with cells grown in reference state (tryptose-yeast extract-glucose medium). Riboflavin biosynthesis protein, ornithine carbamoyltransferase, cystathionine beta-lyase, and threonine dehydratase were the predominant proteins that exhibited 2.19 to 8.5 fold increase in the expression level in cells growing on CMM.

**Conclusion:**

Ornithine carbamoyltransferase and cystathionine beta-lyase were over-expressed in cells grown on cooked meat medium and also identified in the surface protein fraction and the former was immunogenic; making them potential vaccine candidates. Based upon bioinformatic analysis; choloylglycine hydrolase family protein, cell wall-associated serine proteinase, and rhomboid family protein were predicted as surface protein markers for specific detection of *C. perfringens *from the environment and food. Most of the proteins over-expressed in CMM were shown to have putative function in metabolism, of which seven were involved in amino acid transport and metabolism or lipid metabolism.

## Background

*Clostridium perfringens *is a medically important clostridial pathogen and an etiological agent, causing several diseases in humans and animals; the former includes gas gangrene, food poisoning, necrotizing enterocolitis of infants and enteritis necroticans [1-3]. It is an obligate anaerobic rod-shaped bacterium commonly found in the gastrointestinal tracts of both animals and humans and widely distributed in soil and sewage. The ability of *Clostridium perfringens *to cause disease is associated with the production of a variety of extracellular toxins (13 different toxins have been reported so far). On the basis of differential production of toxins, the strains of *C. perfringens *can be divided into five types A through E [3]. Type A strains cause gas gangrene, the most destructive of all diseases, which is characterized by rapid destruction of tissue with production of gas. The incidence of disease ranged from 1% or less of wounded personnel during World War II to 10% of wounded personnel during World War I. Hundreds of thousands of soldiers died of gas gangrene as a result of battlefield injuries, and *C. perfringens *was widely recognised as being the most important causal organism of the disease. Moreover, *C. perfringens *and its toxins have been listed as potential biological and toxin warfare (BTW) agents and warrants attention towards developing strategies pertaining to detection and protection.

Interest in vaccine against gas gangrene has been intermittent with most effort during the World Wars I and II and devoted to the therapeutic use of antisera. Such antisera raised against toxoids of all of the five species of clostridia associated with gas gangrene were shown to have benefits if the serum was given soon after trauma [4]. Active immunization against the disease has received little attention until a few years back [5-7]. Many of the earlier studies used formaldehyde toxoids but due to inherent problems associated with these preparations, the subsequent studies employed genetic approaches. Immunization with isolated C-domain (CPA247–370) of alpha toxin has shown both, high level circulating antibodies and protection of mice against as high as 50 LD_50 _dose of the toxin [7]. Apart from description of antibody responses to well known *C. perfringens *alpha toxin, responses to non-toxin antigens have been little explored. A number of clinical studies in other pathogenic bacteria including *C. difficile *have highlighted the importance of non-toxin protein antigens in disease expression [8-11]. For instance, *C. difficile *surface layer protein (SLP) has been shown to contain antigenic epitopes and play role in colonization of the bacterium to gastrointestinal tissues [8,10].

Complete genome sequences for three of its widely studied strains; *C. perfringens *strain 13, *C. perfringens *ATCC 13124^T ^(a gas gangrene isolate and the species type strain), and *C. perfringens *SM101 (enterotoxin-producing food poisoning strain) have been recently determined and compared [12,13]. Several striking findings have emerged from the complete genome sequencing data of this clostridial pathogen. Comparisons of the three genomes have revealed considerable genomic diversity with >300 unique "genomic islands" identified and using PCR based analysis it was also demonstrated that the large genomic islands were widely variable across a large collection of *C. perfringens *strains [12].

Proteome maps of sequenced organism are important research tools for the authentication of hypothetical proteins, the identification of components of different cellular proteome fractions and for yielding information concerning the occurrence and abundance of proteins. Such proteome maps in the public domain have been generated for many pathogens and are of great value in identifying new virulence factors and the antigens of potential diagnostic and/or curative value against infections with pathogens. Despite a sudden spurt of activity towards proteomic characterization of bacterial pathogens, for reasons unknown, clostridia have largely been ignored. *Clostridium difficile *is the only clostridial species for which analysis of proteome has been carried out to some extent [8,10,14].

To invade, multiply and colonize tissues of the host, a pathogen must be able to evade the host immune system, and obtain nutrients essential for growth. The factors involved in these complex processes are largely unknown and of crucial importance to understanding microbial pathogenesis. Growth of microorganisms *in vitro*, under conditions which mimic certain aspects of the host environment, such as temperature [15], pH [16], nutrient conditions, and interaction with host derived cells [17], can provide valuable information on microbial pathogenesis. Proteome analysis is one of the best tools for understanding the basic biology of microorganisms including pathogenesis, physiology, and mechanisms of avoiding host immune system.

In this study we report identification of major surface and cell envelope proteins from *Clostridium perfringens *ATCC13124 and those differentially expressed in cells grown on cooked meat medium (CMM) in comparison with cells grown in reference state TPYG (tryptose-yeast extract-glucose) medium. Cooked meat medium [18] provides substrate in the form of muscle tissue, for the myonecrotic cells of *C. perfringens *which produces phospholipase C as one of its major virulence factor. Most cases of gas gangrene are associated with an initial focus of infection which develops into a rapidly spreading disease with active invasion of surrounding connective or muscle tissue.

## Results

### Protein identification

**Figure 1:**
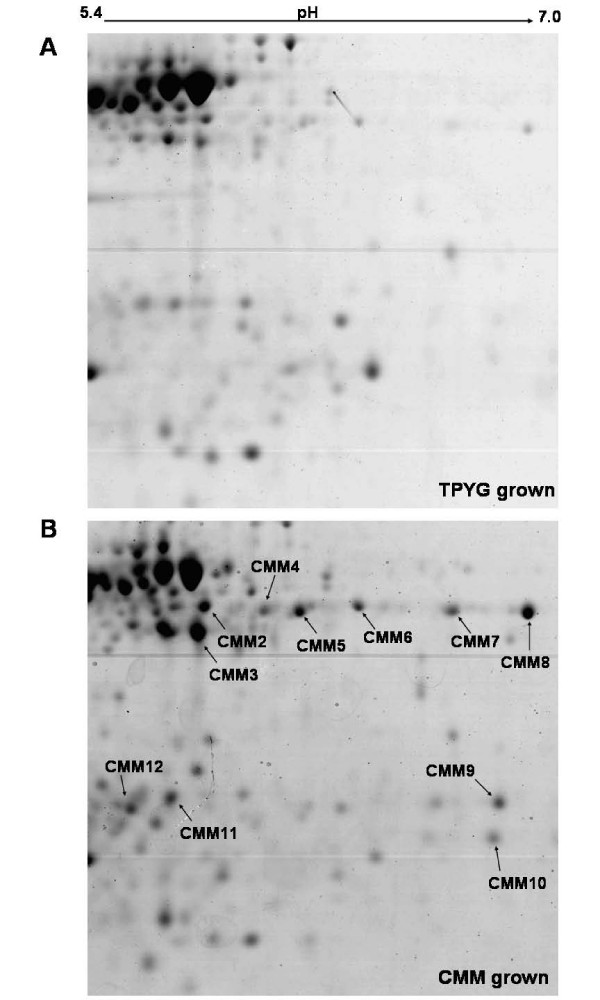
**A portion of representative 2DE gel showing spots quantitatively over-expressed (>2-fold difference) in CMM grown cells (B) of *C*. *perfringens *ATCC13124 as compared to those grown in TPYG medium (A)**. The spots identified are marked with arrows.

**Figure 2:**
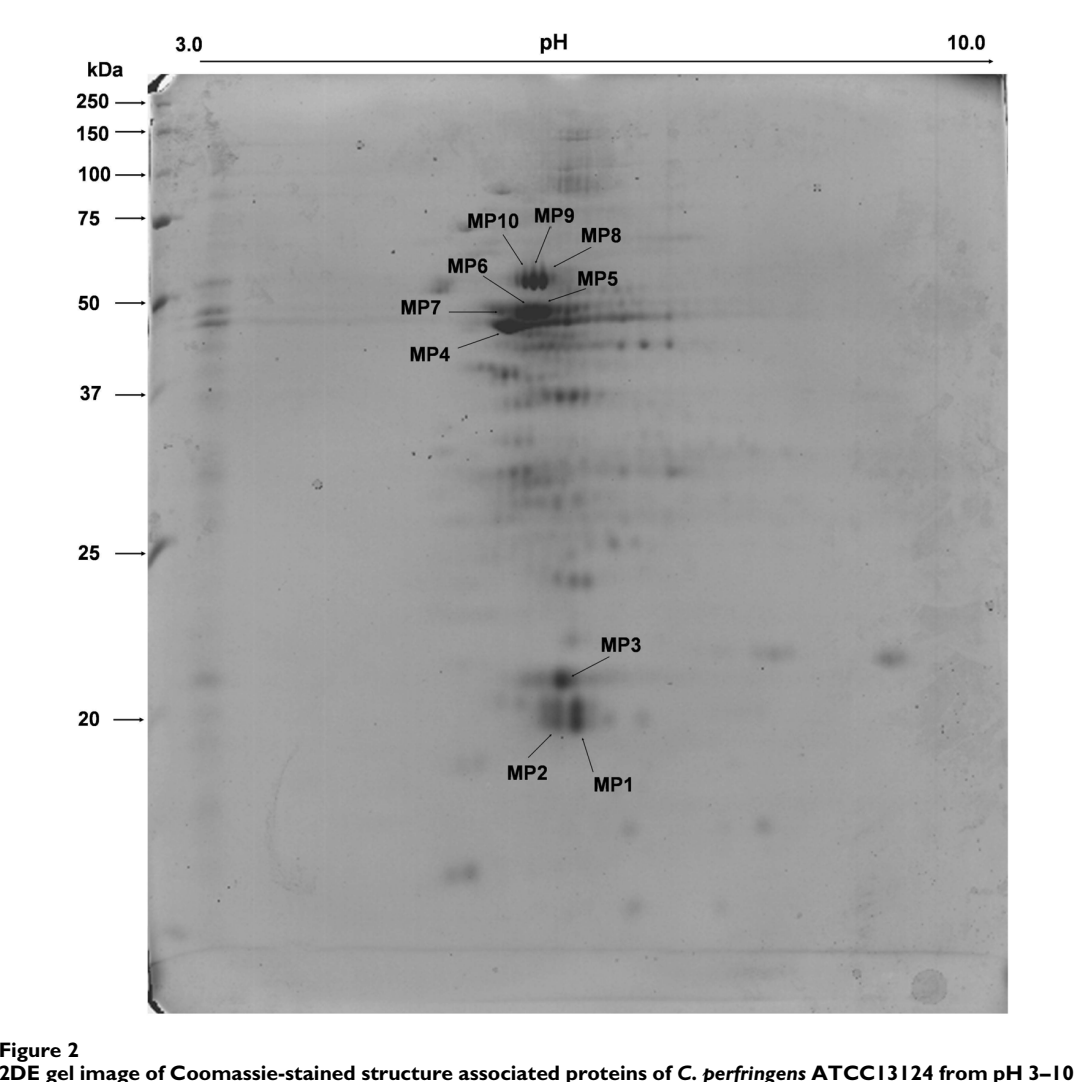
**2DE gel image of Coomassie-stained structure associated proteins of *C*. *perfringens *ATCC13124 from pH 3–10 (17 cm IPG strip)**. Spots identified are indicated with arrows.

**Figure 3:**
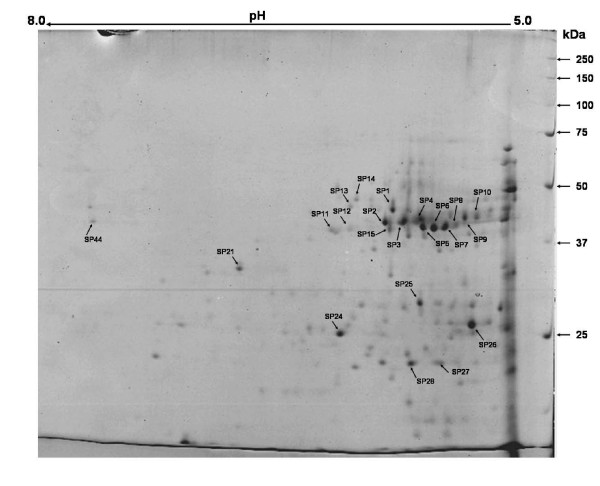
**2DE gel image of Coomassie-stained surface proteins of *C*. *perfringens *ATCC13124 from pH 5–8 (17 cm IPG strip)**. Spots identified are indicated with arrows.

A total of 43 dominant protein spots in three gels (Figure 1, 2, and 3) were marked and analyzed after in gel digestion with trypsin using MLDI-TOF-MS and/or ESI-MS/MS [see Additional file 1 and 2]. This included 22 surface associated proteins, 10 cell envelope proteins, and 12 CMM specific differentially expressed proteins. The gels were analyzed quantitatively to determine the relative abundance of spots and also the fold difference of expression in CMM specific proteins. Since our protein identification was based on ion search at NCBI nonredundant database in the taxonomic group of Bacteria (1348868 entries) or Firmicutes (258665 entries), chances of false positive hits are substantially reduced.

We estimated the MW and p*I *values of the protein spots on the 2-DE gels and compared them with theoretical MW and p*I *values of corresponding proteins from *C. perfringens *ATCC13124. Most of the experimental values matched well with theoretical values, indicating unambiguous identification [see Additional file 1]. Any discrepancies between experimental and theoretical masses might have been caused by post-translational proteolytic processing and modification. The differences between the two p*I *values might be attributed to the cleavage of alkaline regions and phosphorylation of multiple residues.

### CMM induced changes in total cellular protein profile

Figures 1A and 1B show a portion of 2-DE gels of total cellular protein from *C. perfringens *ATCC13124 cells, grown on TPYG and CMM, respectively. The analytical and biological replicates (2 each) of the corresponding 2-DE gels are shown in Additional file 3 and 4. Growth on CMM resulted in over expression of several proteins of which 11 most prominent ones have been identified. To identify the up-regulated proteins, the spots (numbered CMM2-CMM12 in Figure 1) were excised from the gel, digested with trypsin and subjected to MS/MS analysis as detailed in methods. Riboflavin biosynthesis protein, ornithine carbamoyltransferase, cystathionine beta-lyase, and threonine dehydratase were the predominant proteins that exhibited 2.19 to 8.5 fold increase in the expression level in cells grown on CMM (see Additional file 1, Figure 1). Other over expressed proteins include butyryl-CoA dehydrogenase, UDP-glucose 4-epimerase, and electron transfer flavoprotein, showing almost qualitative change in expression (>10 fold) in CMM grown *C. perfringens *cells.

Ornithine carbamoyltransferase (spot CMM3) (see Additional file 1, Figure 1) was the most abundant of the over-expressed proteins and has also been identified in the surface protein fraction of this bacterium (spot SP15) (see Additional file 1, Figure 3). Similarly, cystathionine beta-lyase (spot CMM4) showing 8.5-fold difference of expression in CMM-grown cells of *C. perfringens *was also observed as a dominant cell surface protein (spot SP12) of the bacterium. Curiously, almost all the proteins over-expressed in CMM grown cells were shown to have putative function in metabolism, of which seven were involved in amino acid transport and metabolism or lipid metabolism.

### Cell surface and envelope proteins

A total of 22 surface-localized proteins and 10 cell envelope proteins were identified by proteomic analysis of *C. perfringens *ATCC13124 (see Additional file 1, 2 and 3). For six of the surface proteins the identification was based on MS/MS analysis of the trypsin digested protein, in addition to sequencing of one or more peptides; the independent datasets resulted in same protein match in database search [see Additional file 2].

The identified homologs exhibited high amino acid sequence identity (63–74%) with corresponding proteins from *C. perfringens *ATCC13124 [see Additional file 2] as revealed by blastp results. The 2-DE gel pattern and the identification data of the envelope proteins suggest that rubredoxin and ATP synthase F1, alpha and beta subunit existed as multiple electropherotypes (see Additional file 1, Figure 2). Rubredoxin/rubrerythrin (spots MP1, MP2, and MP3) were the most abundant cell envelope associated proteins which is known to exist as multiple homologs in the *C. perfringens *ATCC13124 genome showing different p*I *values. Except for the spot MP4, all the identified proteins were assigned to the COG functional category of energy production and conversion.

Triosephosphate isomerase, phosphoglycerate kinase, glutamate synthase (NADPH), cell wall-associated serine proteinase, and sucrose-6-phosphate dehydrogenase were the major components in the surface protein fraction of the *C. perfringens *strain (see Additional file 1, Figure 3). Charge variants of aminopeptidase, cystathionine beta-lyase, and translation elongation factor P were some other surface proteins identified. When searched against COG database, most of the dominant surface proteins were predicted to be involved in amino acid transport and metabolism (31.8%), carbohydrate transport and metabolism (18.2%), and translation, ribosomal structure and biogenesis (18.2%).

### Immunogenic proteins

Choloylglycine hydrolase family protein (SP2), glutamate synthase (SP3), sucrose-6-phosphate dehydrogenase (SP4), and ornithine carbamoyltransferase (SP15) were found to be immunogenic cell surface proteins as revealed by the western blot analysis of 2-DE separated surface proteins probed with mouse anti-CPWC serum [see Additional file 5]. We could not identify a few other immunogenic surface proteins visible on western blot.

**Figure 4:**
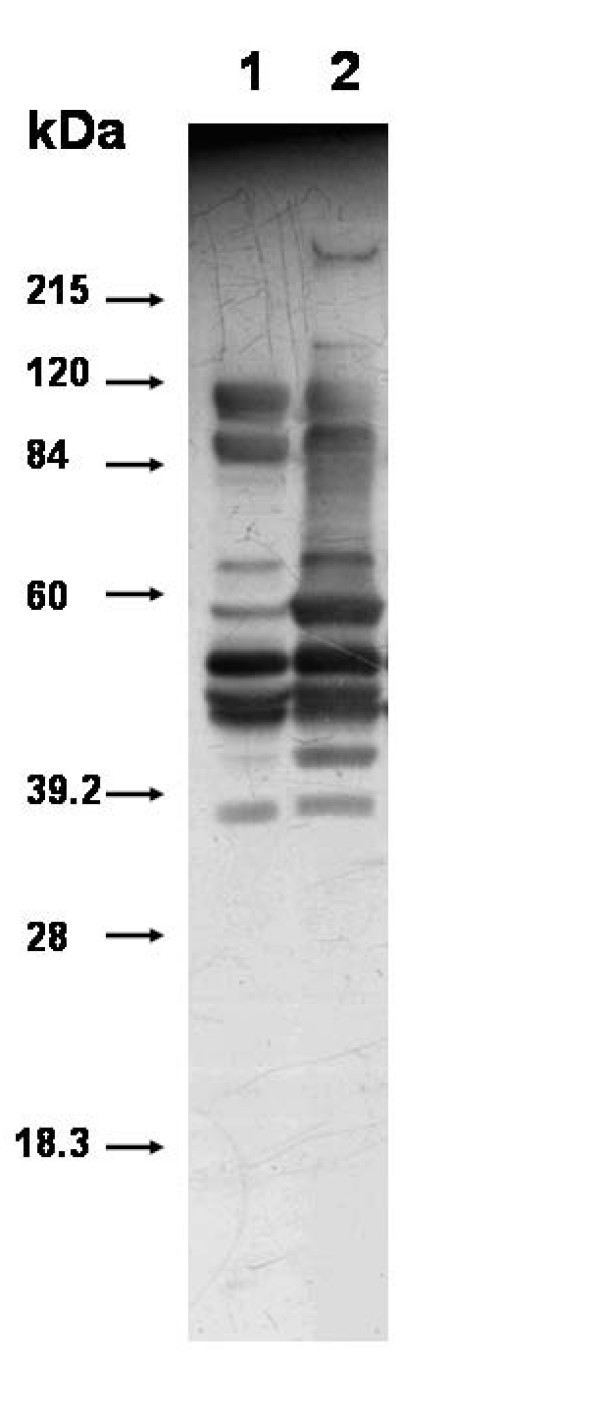
**Western blot analysis of immunogenic proteins of whole cell lysate of *C*. *perfringens *grown on TPYG (lane 1) and CMM (lane 2)**. Protein was separated on 12% SDS-PAGE and transferred onto PVDF membrane. Mouse anti- *C. perfringens *serum (obtained from animals that survived experimental gas gangrene infection) was used to probe the blot and bound antibodies were detected by Goat anti-mouse IgG HRP conjugate by chemiluminescence using and ECL western blot kit (Sigma).

*C. perfringens *ATCC13124 cells were grown on CMM and TPYG till late exponential phase and equal amount of whole cell lysate was separated on one dimensional SDS-PAGE. Western blot was generated using polyclonal serum from mice surviving gas gangrene infection (Figure 4); highlighting proteins recognized by antibodies from *C. perfringens *infected mice. Remarkable differences were observed in the profile of immunogenic proteins, especially in the regions corresponding to molecular masses of 40–42 kDa and 58–60 kDa.

### Sequence analysis of identified proteins

Based on blast search results, all the proteins identified in the present investigation appeared to be highly conserved (showing 94–100% amino acid identity and 97–100% amino acid similarity) among *C. perfringens *strains and were not strain specific (based on whole genome sequence data for 8 strains available in database) [see Additional file 6]. Most of the proteins (32%) were also conserved among other clostridial members showing >70% amino acid sequence identity. Sucrose-6-phosphate dehydrogenase, threonine dehydratase, and N-acetylmuramoyl-L-alanine amidase exhibited 50–60% sequence identity while choloylglycine hydrolase family protein, cell wall-associated serine proteinase, and rhomboid family protein shared only <50% identity with their closest homologs in bacterial domain.

All the identified proteins were analyzed using various bioinformatics software programs, such as SignalP, SecretomeP, PSORT, LipoP, TMHMM, and PROSITE for predicting protein secretion and localization. For instance, N-acetylmuramoyl-L-alanine amidase and cell wall-associated serine proteinase obtained from cell surface fraction of strain ATCC13124 were predicted by SignalP to be secreted in the classical Sec pathway, which is characterized by the presence of a signal peptide [19] [see Additional file 7]. Both these proteins containing the signal peptides possessed cleavage site for signal peptidase 1 (spI). Interestingly, cell wall-associated serine proteinase was also predicted; to harbor two transmembrane helices (TMHMM), suggesting an extracytoplasmic but cell-associated location; contain an LPxTG motif (PROSITE scan) for cell wall anchorage; and a cell wall associated localization (PSORT). PSORT algorithm predicted most of the proteins (49%) to have cytoplasmic localization.

Choloylglycine hydrolase family protein, cell wall-associated serine proteinase, rubredoxin/rubrerythrin, and rhomboid family protein were predicted by SecretomeP to be secreted by non-classical sec pathway characterized by the lack of typical export signals [20].

## Discussion

Cooked meat medium was developed by Robertson [18] in 1916 for use in the cultivation of certain anaerobes isolated from wounds. The present formulation for CMM is a modification of Robertson's original formula. Cooked Meat Medium is still widely used for the cultivation and maintenance of clostridia and the medium is recommended for use in the enumeration and identification of *Clostridium perfringens *from food [21]. Cooked Meat Medium provides a favorable environment for the growth of *C. perfringens*, since the muscle protein in the heart tissue granules is a source of amino acids and other nutrients. The muscle tissue also provides reducing substances, particularly glutathione, which permits the growth of strict anaerobes [22].

The combination of 2-DE and MS has clearly identified major proteins over-expressed in cells of *C. perfringens *ATCC13124 when grown on CMM. We have identified eleven prominent proteins showing over expression CMM grown whole cell proteome of *C. perfringens *ATC13124 cells (see Additional file 1, Figure 1).

For a bacterial protein to be considered as a candidate vaccine antigen, it should preferably be conserved (i.e. present in all strains), secreted or surface localized, and immunogenic (i.e. capable of stimulating the immune system). Ornithine carbamoyltransferase (cOTC) was an abundant protein up-regulated in CMM-grown cells. It was also identified as an immunogenic surface protein of this bacterium (spot SP15) (see Additional file 1 and 5, Figure 3). In another study, ornithine carbamoyltransferase has been isolated as putative adhesin from surface molecule preparation of *Staphylococcus epidermidis *[23]. cOTC is a bonafied cell wall protein of *Streptococcus agalactiae *[24], *S. pyogenes *[25], *S. sanguis *[26], and *S. suis *[27]. Taken together, this makes cOTC a putative vaccine candidate against *C. perfringens *infection. Similarly, cystathionine beta-lyase (spot CMM4) that was over-expressed in CMM-grown cells of *C. perfringens*, has been previously shown as a dominant cell surface protein of the bacterium, indicating a possible role of this protein in pathogenesis and a potential as putative vaccine candidate. Electron transfer flavoprotein, over-expressed in CMM grown cells has been recognized in earlier studies as cross reactive protein of *C. tetani *when probed with mouse anti *C. perfringens *(heat killed organism) polyclonal serum [28] and also as an extracellular protein in *Bacillus anthracis *[29] and *Mycobacterium tuberculosis *[30].

Antibodies from animals surviving gas gangrene infection recognized proteins from both TPYG and CMM grown cells of *C. perfringens *ATCC13124 (Figure 4), though remarkable differences were observed in the profile of immunogenic proteins, especially in the regions corresponding to molecular masses of 40–42 kDa and 58–60 kDa. Curiously, six proteins in the molecular mass range of 40–42 kDa have also been shown to be over-expressed in *C. perfringens *ATCC13124 cells when grown on CMM, using 2-DE profiling of whole cell proteins. These proteins varied in their observed p*I *values from 5.6 – 7.0 and are likely to migrate closely on a one dimensional SDS-PAGE. The results indicate that with reference to TPYG grown cells, some additional proteins expressed *in vivo *(in mouse experimental gangrene model) are also expressed when *C. perfringens *ATCC13124 cells are grown on CMM. Based on the results obtained in the present investigation, it will be highly speculative to suggest that CMM provides host simulated conditions for *C. perfringens*. In a pre-gangrenous infection, *C. perfringens *cells encounter live muscle and immune cells that will be responding and fighting to kill the bacterium. By comparison, cooked meat media (CMM) is processed, granulated and boiled muscle tissue. Further work using proteome from cells obtained from infected host and those from CMM and TPYG grown cells may provide further clue in this direction.

Most of the cell envelope and up-regulated proteins existed as multiple isoelectropherotypes and often differences in their observed and theoretical p*I *values were more pronounced, compared to those observed for molecular masses [see Additional file 1]. We cannot exclude a possibility that there are major post translational events in these proteins resulting in p*I *value differences. Nevertheless, earlier observations have indicated that different isoelectropherotypes of polypeptides in 2-DE gels do not always arise from true post translational modifications, but also from the 2-DE procedure itself [31,32].

The outer surface of bacteria is of great importance to the understanding of bacterial pathogenesis. Elements of the surface are implicated in bacterial defense mechanisms and virulence related functions e.g. adhesion, invasion, direct injury, and induction of septic shock. There is no information available with respect to surface proteins of this medically important bacterium. In the present study, several of the surface proteins and those over-expressed in CMM grown cells were largely assigned putative function in amino acid transport and metabolism [see Additional file 1], suggesting that this organism is adapted to protein rich environment of host tissue. Together, these identified and predicted proteins could be useful targets for the development of improved vaccines against gangrenous infections.

Two of the surface proteins of *C. perfringens*, ornithine carbamoyltransferase and phosphoglycerate kinase have also been identified as immunogenic proteins in the outer surface protein preparation of *S. agalactiae *and *S. pyogenes *[24,25]. Curiously, sera directed against the two proteins were shown to protect neonatal animals from *S. agalactieae *infection in a passive immunization experiment. In earlier studies, phosphoglycerate kinase was reported on the surface of *S. pneumoniae*, was antigenic in humans, and elicited protective immune responses in mouse model [33] [see Additional file 6]. Also in *Schistosoma mansoni*, phosphoglycerate kinase has been identified as a protective antigen [34]. Another surface protein, EF-G, identified in this study was found to be immuno-reactive against sera from broiler chicken immune to necrotic entritis [30]. The protein was secreted into the culture supernatant and unique to virulent *C. perfringens *strain CP4 causing necrotic entritis. Notably, EF-G is regulated by the VirR-VirS virulence regulon of *C. perfringens *[35]. Moreover, EF-G has been demonstrated as an immunogenic protein and was identified in both cell surface and extracellular fraction of *B. anthracis *[9,29].

Further, choloylglycine hydrolase family protein, cell wall-associated serine proteinase, and rhomboid family protein can be excellent surface protein markers for specific detection of *C. perfringens *from environment and food as they share very low percent amino acid sequence identity with there nearest homologs (<50%) and are conserved among the *C. perfringens *strains [see Additional file 6].

Some of the surface proteins from *C. perfringens *ATCC13124 showed metabolic functions that would typically place them in the cytoplasm. Moreover, except for N-acetylmuramoyl-L-alanine amidase and cell wall-associated serine proteinase, these proteins have no N-terminal signal peptide and do not possess the canonical gram-positive anchor motif LPXTG [see Additional file 7]. Several surface-associated cytoplasmic proteins reported in this study were also detected on the bacterial surface in previous proteomic analysis [see Additional file 6]. For example, phosphoglycerate kinase was reported on the surface of *S. pneumoniae *[33], *S. agalactiae *[24], *S. pyogenes *[25], and *S. oralis *[see Additional file 6] and also as secreted protein in *B. anthracis *[29]. Increasing number of reports have shown presence of proteins on the surface of Gram positive bacteria or secreted into the medium that one would otherwise expect to be cytoplasmic [25,29,36,37]. In a previous study, the culture supernatant of *C. perfringens *at the late exponential growth phase was shown to contain intracellular proteins that had no putative signal sequences, such as ribokinase, β-hydroxybutyryl-coenzyme A dehydrogenase, fructosebisphosphate aldolase, and elongation factor G [36]. In other studies also, a significant number of cytoplasmic proteins have been identified as cell-wall associated proteins/immunogens [25,37].

In spite of a growing list of cytoplasmic proteins identified on the bacterial surface, the mechanism of their surface localization and attachment to the bacterial envelope remain unclear. Internal signal sequences, posttranslational acylation, or an association with a secreted protein are hypothesized as possible means [38]. The mapping of proteins to the bacterial surface may well suggest alternate functions for proteins with other well established roles. For instance, the glycolytic enzyme α-enolase has been shown as plasmin-binding protein on the outside of the bacterial cells [38].

For most of the cell envelope proteins identified here, a surface localization cannot be ruled out as not all of the proteins from the cell surface fraction could be identified. The translation elongation factor Tu (spot MP4) has been shown to be surface associated protein in *S. pyogenes *[25,39] and other Gram-positive bacteria [40-42]. Little is known about the possible functions of surface-associated elongation factors on the bacterial surface. Nevertheless, elongation factor of *Lactococcus johnsonii *is shown to be involved in attachment of this pathogen to human intestinal cells and mucins [40], while the same protein in *Mycobacterium pneumoniae *binds fibronectin, which mediates the attachment of pathogen to host cells [43]. It has also been reported as immunogenic spore protein of *Bacillus anthracis *[9] and a virulence determinant in *Coxiella burnetii *[44].

## Conclusion

Eleven prominent proteins showing over expression on CMM grown cells using whole cell proteome of *C. perfringens *ATC13124 have been identified by 2-DE MS approach. In addition the predominant cell surface and cell envelope (structure associated) proteins were also identified and a few were found to be common with those observed as over-expressed in CMM grown cells. Cystathionine beta-lyase and Ornithine carbamoyltransferase identified in this study can be putative vaccine candidates as they are over-expressed in CMM grown cells, are surface localized, the latter is immunogenic, and their homologs in other pathogenic bacteria have been shown to be immunogenic/virulence factor. In addition phosphoglycerate kinase, N-acetylmuramoyl-L-alanine amidase, and translation elongation factor Tu and EF-G can also be putative vaccine candidates as they are abundant on the cell surface fraction and their homologs in other Gram positive pathogenic bacteria have been shown to be immunogenic/virulence determinants. We propose choloylglycine hydrolase family protein, cell wall-associated serine proteinase, and rhomboid family protein as potential surface protein markers for specific detection of *C. perfringens *from environment and food.

## Methods

### Bacterial strain and growth conditions

*Clostridium perfringens *ATCC13124 was obtained from Becton Dickinson India Pvt. Ltd., India. The bacterium was cultivated anaerobically at 37°C in TPYG broth containing pancreatic digest of casein, 50 g; peptone, 5 g; yeast extract, 20 g; glucose, 4 g; sodium thioglycollate, 1 g; cycloserine, 250 mg; sulphamethoxazole, 76 mg and trimethoprim, 4 mg per litre. The strain was grown under experimental conditions on cooked meat medium (CMM) containing beef heart granules, 454 g; proteose petone, 20 g; dextrose, 2 g; sodium chloride, 5 g per litre. The pH was maintained at 7.0 – 7.5 and agar was added to a final concentration of 2% for preparation of solid media. The inoculation was carried out in an anaerobic workstation (Don Whitley Scientific Ltd., Shipley, England) operating at 37°C. The anaerobic gas mixture was composed of 85% N_2_, 10% H_2 _and 5% CO_2_. The plates were then transferred into anaerobic gas jar (Oxoid Ltd., England) containing palladium catalyst and a gas generation kit (Oxoid Ltd., England) as per manufacturer's instructions.

### Immunization and preparation of polyclonal sera

Animal experiments were approved by the institutional Animal Ethical Committee at DRDE, Gwalior. For probing immunogenic surface proteins, polyclonal serum was generated as follows. Four-week-old female BALB/c mice were actively immunized against heat-killed vegetative cells of *C. perfringens *in a four week immunization schedule. Cells were grown in TPYG broth at 37°C, harvested in the exponential phase (OD_600 nm _0.8–1.0) and washed with phosphate buffer saline (PBS). The number of bacteria in the final suspension was determined by plating 10-fold serial dilutions onto SPS agar (Difco, USA) plates containing tryptone, 15 g; yeast extract, 10 g; ferric citrate, 0.5 g; sodium sulfite, 0.5 g; sodium thioglycollate, 0.1 g; polysorbate 80, 0.05 g; sulfadiazine, 0.12 g; polymyxin B sulfate, 0.01 g; agar, 15 g per litre. Heat inactivation was accomplished in a water bath at 60°C for 30 min. No live bacteria were detected after this suspension was plated onto agar plates. Cells were injected intraperitoneally using Freund's complete adjuvant (Sigma Aldrich, India) for the first immunization and Freund's incomplete adjuvant for booster immunizations. On day 1 and 7, 10^2 ^cfu (100 μl cell suspension in PBS and 100 μl adjuvant) was injected in each mouse while on day 14 and 27 the dose was increased to 10^4 ^cfu. One week after administration of the last booster, 10 animals were anesthetized by halothane inhalation, and blood specimen (500 μl) was obtained from each by means of retro-orbital puncture. Serum from these specimens was pooled and was used for Western blot analysis of surface proteins. Sham-immunized animals received an equal volume of adjuvant alone in a parallel, same immunization schedule and serum was collected after 5 weeks.

For probing whole cell lysate from CMM and TPYG grown cells, polyclonal serum from mice surviving gas gangrene infection was obtained as follows. *C. perfringens *ATCC13124 cells were grown in TPYG broth at 37°C and harvested in exponential phase. Four-week-old female BALB/c mice in groups of 6 each were given intramuscular injection of 10^6^, 10^7^, 10^8 ^and 10^9 ^CFU of washed *C. perfringens *cells in a volume of 0.1 ml anaerobically prepared saline into the right hindquarter through a 26-gauge needle [45]. Mice infected with 10^8 ^and 10^9 ^CFU of *C. perfringens *cells developed swollen hemorrhagic thighs and 3 of those receiving 10^8 ^cells, survived infection. Serum was collected after 4 weeks of inoculation from all the mice that developed gas gangrene and survived.

### Preparation of whole cell protein extract

For differential proteomic analysis, *C. perfringens *ATCC13124 was anaerobically grown on TPYG and CMM agar at 37°C for 24 hrs (corresponding to stationary phase of growth) and the surface growth was harvested using 50 mM Tris/HCl, pH 7.2. Care was taken to avoid contamination from agar medium and the cells were washed in 50 mM Tris/HCl, pH 7.2. The cells were resuspended in the same buffer supplemented with protease inhibitor (Protease inhibitor cocktail, Sigma). Cell lysis was performed by sonication and the un-disrupted cells were removed by centrifugation (10000 × g; 15 min; 4°C).

### Preparation of cell surface and cell envelope protein

Cell surface protein was prepared by the method reported earlier for another Gram positive bacterium [46]. Briefly, *C. perfringens *cells were grown on TPYG broth at 37°C and twenty milliliter of culture was harvested in the exponential growth phase (OD_600 nm_~0.8). The harvested cells were washed twice with pre-cooled 50 mM Tris-HCl buffer, pH 7.2 and resuspended in 50 mM Tris-HCl buffer, pH 7.2 containing 2% (w/v) CHAPS. The protein preparation was placed on ice for 2 h, followed by centrifugation at 3500 × g at 4°C for 30 min to separate the cell surface proteins. The supernatant was filtered through a 0.22 μm syringe filter (Milipore, India) to obtain a cell free surface protein preparation.

For preparation of cell envelope (structure-associated) protein, the cells were grown on TPYG broth at 37°C and twenty milliliter of culture was harvested in the exponential growth phase (OD_600 nm_~0.8). The harvested cells were washed twice with pre-cooled 50 mM Tris-HCl buffer, pH 7.2 and resuspended in the same buffer. Cell lysis was performed by sonication and the un-disrupted cells were removed by centrifugation (10,000 × g; 15 min; 4°C). Cell envelope proteins were then collected by centrifugation (40,000 × g; 30 min; 4°C) and washed three times with distilled water. The pellet was resuspended in distilled water, divided into aliquots and stored at -80°C until use.

Total protein concentration was determined according to the method of Bradford [47] using Quick Start Bradford Protein Assay kit (Bio-Rad, USA) as per manufacturer's instructions. The protein concentration was calculated using bovine serum albumin (BSA) as standard.

### 2-DE

In order to improve focusing, proteins samples were purified using 2D-cleanup kit (Bio-Rad) and the protein pellet was finally resuspended in sample rehydration buffer (8 M urea, 2% w/v CHAPS, 15 mM DTT and 0.5% v/v IPG buffer pH 3–10).

The isoelectric focusing was performed using immobilized pH gradient (IPG) strips (Bio-Rad, USA). IPG strips with a pH range from 5–8 were used for all the experiments except for the separation of surface proteins where strips of pH range 3–10 were used. For the first dimension 500 μg of protein samples in 300 μl of rehydration solution was used to rehydrate IPG strip (17 cm, pH 5–8). The IPG strips were rehydrated overnight and then the proteins were focused for 10000 VHr at 20°C under mineral oil. After focusing, the strips were incubated for 10 min, in 4 ml of equilibrium buffer I (6 M urea, 30% w/v glycerol, 2% w/v SDS and 1% w/v DTT in 50 mM Tris/HCl buffer, pH 8.8) followed by equilibrium buffer II (6 M urea, 30% w/v glycerol, 2% w/v SDS and 4% w/v iodoacetamide in 50 mM Tris/HCl buffer, pH 8.8). After the equilibration steps the strips were transferred to 12% SDS-PAGE for the second dimension by the method of Blackshear [48]. Protein spots were visualized by staining with Coomassie Brilliant Blue G-250. Gel images were captured by GS800 densitometer (Bio-Rad, USA). Relative abundance of the spots and the differential protein expression were determined by PD Quest software (Bio-Rad, USA). Two independent experiments were carried out for the differential study and replicate gels were generated from each independent experiment.

### Immunoblotting

For immunoblotting of whole cell proteins obtained from TPYG and CMM grown cells, the SDS-PAGE separated proteins on one dimension were transferred electrophoretically to PVDF membrane (Bio-Rad, Hercules, CA) and then blocked with PBS (pH 7.2) containing 5% nonfat dry milk and 0.05% Tween 20. Serum obtained from mice surviving *C. perfringens *infection was used at 1:1000 dilutions in blocking buffer. Goat anti-mouse HRP conjugate (Dako) was used as secondary antibody at 1:30000 dilutions. Bound antibodies were detected by chemiluminescence using an ECL western blot kit (Sigma) and Hyperfilm ECL (Amersham) as per manufacturer's instructions. Film was exposed for 15 sec before development.

For analysis of immunogenic surface proteins, Goat anti-mouse HRP conjugate was used as secondary antibody (1:2000 dilutions) and blots were developed using Immuno-Blot HRP assay kit (Bio-Rad, USA) as per manufacturer's instructions.

### Identification of protein spots by mass spectrometry

Protein spots were excised with the help of thin-walled PCR tubes (200 μl) appropriately cut at the bottom with the help of fresh surgical scalpel blade. Care was taken not to contaminate the spots from adjoining proteins or with skin keratin. The gel spots were washed with proteomic grade de-ionized water and proteins identified by mass spectrometry by the commercial services provided by Proteomics International Pty Ltd., Australia and The Centre for Genomic Application, India.

The gel piece containing the protein was destained, reduced/alkylated and trypsin digested using the Montage In-Gel Digest Kit (Millipore) following the kit's instructions. For cell envelope proteins, peptides were analyzed by electrospray time-of-flight mass spectrometry (LC/MS/TOF) using a QStar Pulsar i (Applied Biosystems). Spectra were analyzed using Mascot sequence matching software from Matrix Science (http://www.matrixscience.com using non identical protein sequence database based on MSDB in the taxonomy group of Bacteria. Search parameters were: maximum of one missed cleavage by trypsin, fixed modification of oxidation, charged state of +1, and fragment mass tolerance of ± 0.6 Da.

MALDI-TOF-TOF system from Bruker Daltonik and ESI-MS/MS from Agilent 1100 series 2DnanoLC MS, were used for the analysis of surface proteins and differentially expressed proteins. Identification was carried out using one or more of the MS/MS platforms shown in Additional file 2. Peptide mass fingerprinting data of trypsin digested proteins, combined MS/MS ion of peptides, and MS/MS analysis results of one or more selected peptides were used for database search as described above. In most of the cases, proteins were identified as homologs in other clostridial species closely related with *C. perfringens *[see Additional file 2]. Homology searches were carried out using the BLAST and PSI-BLAST protein algorithm against the GeneBank nonredundant protein database http://www.ncbi.nlm.nih.gov. The theoretical molecular weights and isoelectric points were determined using the Compute p*I*/Mw algorithm http://ca.expasy.org/. Pattern/profile, post translational modifications and topology search were carried out using ExPASy Proteomics tools at http://www.expasy.ch.

## Authors' contributions

SIA designed and executed most part of the experiments including proteomic studies and bioinformatic analysis. SB, RBK, and NS participated in running 2DE gels and immunisation of animals. LS provided supervision of the research group and critically revised the manuscript for its important intellectual content. All authors read and approved the final manuscript.

## Supplementary Material

Additional file 1 **Protein spots identified from surface and cell wall components of *C. perfringens *ATCC13124 and those differentially expressed on cooked meat medium **Summary of protein identification results and relative abundance. Click here for file

Additional file 2 **Proteins identified from *C. perfringens *ATCC13124**. The table reports: 1) the MASCOT top hit, 2) homologous protein in *C. perfringens *ATCC13124 proteome^a ^with percent identity, and 3) the peptides generated by trypsin digestion, the platform for their identification by mass spectrometry and corresponding MASCOT scores. Click here for file

Additional file 3 **Whole cell proteome of *Clostridium perfringens *ATCC13124 grown on cooked meat medium**. Proteins were separated by 2-DE. Approximately 500 μg of total cellular proteins were separated on 17 cm IPG strips (pH 5–8) and stained with Coomassie brilliant blue R250. R1 and R2 are analytical replicates of experiment-1 while R3 and R4 are analytical replicates of experiment 2. Click here for file

Additional file 4 **Whole cell proteome of *Clostridium perfringens *ATCC13124 grown on TPYG medium**. Proteins were separated by 2-DE. Approximately 500 μg of total cellular proteins were separated on 17 cm IPG strips (pH 5–8) and stained with Coomassie brilliant blue R250. R1 and R2 are analytical replicates of experiment-1 while R3 and R4 are analytical replicates of experiment 2. Click here for file

Additional file 5 **Western blot analysis of immunogenic surface proteins from *C. perfringens *ATCC13124**. Surface protein fraction was separated by 2-DE and probed with mouse anti- *C. perfringens *(heat killed whole cell) serum. Goat anti-mouse HRP conjugate was used as secondary antibody (1:2000 dilutions) and blots were developed using Immuno-Blot HRP assay kit (Bio-Rad, USA) as per manufacturer's instructions. **A**, Coomassie stained 2-DE gel; **B**, corresponding blot as described above. Spots identified in this study are indicated with arrows. Click here for file

Additional file 6 **Proteins identified in this study and their homologues in other bacteria**. A few pathogenic organisms where the presence of respective protein has been shown experimentally in other studies are listed along with their localization and predicted role. Click here for file

Additional file 7 **Pattern/profile, post translational modifications and topology search results for identified proteins of *Clostridium perfringens***. Proteins identified from different fractions, indicating theoretical localization. All the analysis was carried out using ExPASy Proteomics tools at http://www.expasy.ch. Click here for file
