# Supplementary material for: Differential proteomic analysis of Clostridium perfringens ATCC13124; identification of dominant, surface and structure associated proteins
Source: BMC Microbiol. 2009 Aug 10;9:162. doi: 10.1186/1471-2180-9-162 (PMC2731776; doi:10.1186/1471-2180-9-162)
Supplement: Additional file 1 — Protein spots identified from surface and cell wall components of C. perfringens ATCC13124 and those differentially expressed on cooked meat medium Summary of protein identification results and relative abundance. [file 1471-2180-9-162-S1.doc]

**Additional file 1:** Protein spots identified from surface and cell wall components of *C. perfringens* ATCC13124 and those differentially expressed on cooked meat medium.

| **Spot #** | **Accession No.** | **Protein name** | **M_r_ / p*I* _theoretical_^a^** | **M_r_ / p*I* _observed_** | **Relative abundance (%)^b^** | **COG (Functional category) ^c^** |
| --- | --- | --- | --- | --- | --- | --- |
|  |  |  |  |  |  |  |
| **A. Surface protein** | | |  |  |  |  |
| SP1 | YP_696206 | Glutamate dehydrogenase | 49/6.01 | 43/5.7 | 42 | COG0334 (E) |
| SP2^d^ | YP_695794 | Choloylglycine hydrolase family protein | 36/5.43 | 42/5.6-5.8 | 58 | COG3049 (M) |
| SP3^d^ | YP_695906 | Glutamate synthase (NADPH), homotetrameric | 42/7.67 | 42/5.6-5.8 | 84 | COG0493 (ER) |
| SP4^d^ | YP_696219 | Sucrose-6-phosphate dehydrogenase, | 57/5.73 | 38-40/5.4-5.6 | 71 | COG1621 (G) |
| SP5 | YP_695953 | Phosphoglycerate kinase | 42/5.29 | 38-40/5.4-5.6 | 53 | COG0126 (G) |
| SP6 | YP_695953 | Phosphoglycerate kinase | 42/5.29 | 38-40/5.4-5.6 | 85 | COG0126 (G) |
| SP7 | YP_695569 | Cell wall-associated serine proteinase | 172/5.02 | 38-40/5.4-5.6 | 75 | COG1404 (O) |
| SP8 | YP_694671 | Acetate kinase | 43/5.58 | 40 / 5.2-5.4 | 47 | COG0282 (C) |
| SP9 | YP_696676 | Aminopeptidase | 46/4.99 | 42/5.2-5.4 | 68 | COG2309 (E) |
| SP10 | YP_696676 | Aminopeptidase | 46/4.99 | 42/5.2-5.4 | 43 | COG2309 (E) |
| SP11 | ABG82803 | Cystathionine beta-lyase | 42/5.48 | 38/6.0-6.2 | 41 | COG0626 (E) |
| SP12 | YP_694633 | Cystathionine beta-lyase | 42/5.48 | 38/5.8-6.0 | 26 | COG0626 (E) |
| SP13 | YP_697056 | N-acetylmuramoyl-L-alanine amidase | 25/8.82 | 45/5.8-6.0 | 18 | COG0860 (M) |
| SP14 | YP_694487 | Seryl-tRNA synthetase | 48/5.37 | 47/5.8-6.0 | 18 | COG0172 (J) |
| SP15^d^ | YP_694626 | Ornithine carbamoyltransferase, | 37/5.40 | 38/5.6-5.8 | 38 | COG0078 (E) |
| SP21 | YP_694753 | Putative transketolase, C-terminal subunit, | 33/6.48 | 31/6.4-6.6 | 33 | COG0021 (G) |
| SP24 | Q8XIR2 | Deoxyribose-phosphate aldolase | 23/5.21 | 25/6.0 | 64 | COG0274 (F) |
| SP25 | Q8XHS1 | Elongation factor G (EF-G) | 77/5.37 | 28/5.4-5.6 | 50 | COG0480 (J) |
| SP26 | Q0TQY8 | Triosephosphate isomerase | 27/5.01 | 26/5.2-5.4 | 100 | COG0149 (G) |
| SP27 | Q0TPC2 | Translation elongation factor P | 20/4.92 | 19/5.6 | 48 | COG0231 (J) |
| SP28 | Q0TPC2 | Translation elongation factor P | 20/4.92 | 20/5.6-5.8 | 31 | COG0231 (J) |
| SP44 | YP_696141 | Rhomboid family protein | 38/8.45 | 40/7.2-7.4 | 23 | COG0705 (R) |
|  |  |  |  |  |  |  |
| **B. Cell envelope protein** | | |  |  |  |  |
| MP-1 | YP_695233 | Rubredoxin, [ABG84689, ABG84965, ABG83958] | 22/5.37  19/5.11  21/5.05 | 20/5.93 | 100 | COG1592 (C) |
| MP-2 | YP_697309 | Rubredoxin/rubrerythrin,  [ABG84689, ABG84965, ABG83958] | 22/5.37  19/5.11  21/5.05 | 20/5.74 | 80 | COG1592 (C) |
| MP-3 | YP_697309 | Rubredoxin/rubrerythrin,  [ABG84689, ABG84965, ABG83958] | 22/5.37  19/5.11  21/5.05 | 21/5.81 | 47 | COG1592 (C) |
| MP-4 | YP_697096,  YP_697110 | Translation elongation factor Tu | 43/4.94 | 48/5.36 | 35 | COG0050 (JE) |
| MP-5 | YP_696866 | ATP synthase F1, beta subunit | 50/4.98 | 50/5.81 | 16 | COG0055 (C) |
| MP-6 | YP_696866 | ATP synthase F1, beta subunit | 50/4.98 | 50/5.62 | 20 | COG0055 (C) |
| MP-7 | YP_696866 | ATP synthase F1, beta subunit | 50/4.98 | 50/5.55 | 37 | COG0055 (C) |
| MP-8 | YP_696868 | ATP synthase F1, alpha subunit | 55/5.28 | 58/5.60 | 30 | COG0056 (C) |
| MP-9 | YP_696868 | ATP synthase F1, alpha subunit | 55/5.28 | 58/5.51 | 29 | COG0056 (C) |
| MP-10 | YP_696868 | ATP synthase F1, alpha subunit | 55/5.28 | 58/5.44 | 18 | COG0056 (C) |
|  |  |  |  |  |  |  |
| **C. CMM specific protein** | | |  |  |  |  |
| CMM2 | YP_695002 | Riboflavin biosynthesis protein | 44/5.42 | 44.0/5.6-5.8 | 5.0 (+2.19)**^e^** | COG0807 (H) |
| CMM3 | YP_694626 | Ornithine carbamoyltransferase, catabolic | 37/5.40 | 42.0/5.6.5.8 | 15.2 (+3.88) | COG0078 (E) |
| CMM4 | YP_694633 | Cystathionine beta-lyase | 42/5.48 | 41.0/5.8-6.0 | 2.8 (+8.50) | COG2873 (E) |
| CMM5 | YP_695814 | Threonine dehydratase, catabolic | 43/6.77 | 41.0/6.0-6.2 | 4.6 (+5.37) | COG1171 (E) |
| CMM6 | YP_695814 | Threonine dehydratase, catabolic | 43/6.77 | 41.0/6.2-6.4 | 3.0 (+8.81) | COG1171 (E) |
| CMM7 | YP_696974 | Butyryl-CoA dehydrogenase | 41/5.65 | 41.0/6.6-6.8 | 3.2 (>10.0) | COG1960 (I) |
| CMM8 | YP_696974 | Butyryl-CoA dehydrogenase | 41/5.65 | 41.0/6.8-7.0 | 5.8 (>10.0) | COG1960 (I) |
| CMM9 | YP_696974 | Butyryl-CoA dehydrogenase | 41/5.65 | 28/6.6-6.8 | 3.8 (>10.0) | COG1960 (I) |
| CMM10 | YP_695002 | Riboflavin biosynthesis protein | 44/5.42 | 31/6.6-6.8 | 3.2 (>10.0) | COG0807 (H) |
| CMM11 | YP_694941 | UDP-glucose 4-epimerase | 37/5.61 | 30/5.6-5.8 | 4.6 (>10.0) | COG1087 (M) |
| CMM12 | YP_696973 | Electron transfer flavoprotein, beta subunit | 27/5.11 | 30/5.5-5.7 | 4.9 (>10.0) | COG2086 (C) |

**^a^** Theoretical values were obtained with the help of an online tool at <http://expasy.org/sprot/>.

**^b^** Relative to most abundant protein on the gel. Spot volume was estimated as average OD x mm^2^ in replicate gels using spot density determination tool in PD Quest software.

**^c^** COGs were assigned after COGnitor search and functional role categories were assigned as per the descriptions in COG page at <http://www.ncbi.nlm.nih.gov/COG>. **E**, amino acid transport and metabolism; **M**, cell envelope biogenesis, outer membrane; **G**, carbohydrate transport and metabolism; **O**, posttranslational modification, protein turnover, chaperones; **C**, energy production and conversion; **J**, translation, ribosomal structure and biogenesis; **H**, coenzyme transport and metabolism; **I**, lipid metabolism; **F**, nucleotide transport and metabolism; **R**, general function prediction only.

**^d^** Immunoreactive to serum raised against *C. perfringens* whole cell in mice.

**^e^** Values in parenthesis indicate fold increase in expression of protein in CMM grown cells calculated taking mean values of three gels.
