# Supplementary material for: Differential proteomic analysis of Clostridium perfringens ATCC13124; identification of dominant, surface and structure associated proteins
Source: BMC Microbiol. 2009 Aug 10;9:162. doi: 10.1186/1471-2180-9-162 (PMC2731776; doi:10.1186/1471-2180-9-162)
Supplement: Additional file 2 — Proteins identified from C. perfringens ATCC13124. The table reports: 1) the MASCOT top hit, 2) homologous protein in C. perfringens ATCC13124 proteomea with percent identity, and 3) the peptides generated by trypsin digestion, the platform for their identification by mass spectrometry and corresponding MASCOT scores. [file 1471-2180-9-162-S2.doc]

**Additional file 2.** Proteins identified from *C. perfringens* ATCC13124. The table reports: 1) the MASCOT top hit, 2) homologous protein in *C. perfringens* ATCC13124 proteome^a^ with percent identity, and 3) the peptides generated by trypsin digestion, the platform for their identification by mass spectrometry and corresponding MASCOT scores.

| **Spot No.** | **MASCOT top hit^b^** | **Peptide sequence** | **Mascot protein score^c^** | **MS/MS platform** | **Homologous protein in strain ATCC13124** | **Percent identity** |
| --- | --- | --- | --- | --- | --- | --- |
| **A. Surface proteins** | | |  |  |  |  |
| SP1 | Glutamate dehydrogenase/leucine dehydrogenase*.- Clostridium* sp. OhILAs, [Q1F0X2_9CLOT](http://www.matrixscience.com/cgi/protein_view.pl?file=../data/20070315/FoTTlzsE.dat&hit=Q1F0X2_9CLOT&px=1&_server_mudpit_switch=0.001) | K.ALSTWMTFK.C  K.ALSTWMTFK.C | 37 | MS/MS  (ESI- TRAP) | Glutamate dehydrogenase,  [YP_696206] | 35 |
|  |  |  |  |  |  |  |
| SP2 | BPU29368 NID: - *Sporosarcina pasteurii*, [AAA73990](http://www.matrixscience.com/cgi/protein_view.pl?file=../data/20070313/FoTpObum.dat&hit=1) | K.DNMGLKK.M  R.LSMEFQYR.G  R.GEVFEYTDVR.T  R.LSMEFQYRGNK.T  K.TVIQMTSDSTFLWGDILSPGR.V  R.SEATVYLVETSGGIVEGDHNVFDIDIK.E  K.MGMLEDHLFIGSWVDTPTIGEFRILGS | 63 | PMF | Choloylglycine hydrolase family protein, [YP_695794] | 45 |
|  |  |  |  |  |  |  |
| SP3 | Putative glutamate synthase [NADPH] small chain *Clostridium difficile* 630, [Q18C58_CLODI](http://www.matrixscience.com/cgi/protein_view.pl?file=../data/20070316/FoTemzue.dat&hit=Q18C58_CLODI&px=1&_server_mudpit_switch=0.001) | K.VAVIGSGPAGLACAGDLAK.K | 76 | SP-3-LCMS-1-A  (ESI-TRAP) | Glutamate synthase (NADPH), homotetrameric,  [YP_695906] | 61 |
|  |  |  |  |  |  |  |
| SP4 | SacA (Glycoside Hydrolase Family 32)- *Bacillus licheniformis*, [Q65DN5_BACLD](http://www.matrixscience.com/cgi/protein_view.pl?file=../data/20070313/FoTufxTS.dat&hit=1) | -.MNQDQELR.Q  R.LPKGYTAHFR.D  K.DGISFEKQGVVAR.L  K.EIIMNVTSWTMA  K.EIIMNVTSWTMA  K.LWGHYSSADLVNWR.H  R.YFPYPGNNDVWISARK.E  R.ETYQCLAVSKDGISFEK.Q  K.KNGSWYMVLGAQTENLEGR.A | 61  (21.3%) | PMF | Sucrose-6-phosphate dehydrogenase, [YP_696219] | 41 |
|  |  |  |  |  |  |  |
|  | Acetate kinase (Fragment).- *Desulfotomaculum reducens* MI-1, [Q2CYH9_9FIRM](http://www.matrixscience.com/cgi/protein_view.pl?file=../data/20070313/FoTuficm.dat&hit=1) | R.YGFHGTSHK.F | 66 | SP-4-MS-MS-1033  (MALDI-TOF-TOF)* | Sucrose-6-phosphate dehydrogenase, [YP_696219] | 100 |
|  |  |  |  |  |  |  |
| SP5 | Phosphoglycerate kinase *Clostridium difficile* 630, [Q181T8_CLODI](http://www.matrixscience.com/cgi/protein_view.pl?file=../data/20070313/FoTpOfcw.dat&hit=Q181T8_CLODI&px=1&_server_mudpit_switch=0.001) | K.MTHVSTGGGASLEFLEGK.E  K.ELASLAEIFVNDAFGTAHR.A  K.FLGEAVANPVRPFTAILGGAK.V | 293 | SP-5-MS-MS-COMBINED  (MALDI-TOF-TOF) | Phosphoglycerate kinase, [YP_695953] | 78 |
|  |  |  |  |  |  |  |
|  | Phosphoglycerate kinase- *Clostridium difficile* 630, [Q181T8_CLODI](http://www.matrixscience.com/cgi/protein_view.pl?file=../data/20070313/FoTpOaYw.dat&hit=1) | R.VCGYLIQK.E  K.VDNLIIGGGMAYTFLK.A  K.VDNLIIGGGMAYTFLK.A  K.MTHVSTGGGASLEFLEGK.E  MTHVSTGGGASLEFLEGK.E  R.CDFNVPLQDGVITDENR.L  K.ELASLAEIFVNDAFGTAHR.A  K.FLGEAVANPVRPFTAILGGAK.V | 68  (24.8%) | PMF | Phosphoglycerate kinase, [YP_695953] | 78 |
|  |  |  |  |  |  |  |
|  | Phosphoglycerate kinase *Clostridium difficile* 630, [Q181T8_CLODI](http://www.matrixscience.com/cgi/protein_view.pl?file=../data/20070313/FoTpOaSt.dat&hit=2) | R.MTHVSTGGGASLEFLEGK.E | 132 | SP-5-MS-MS-1820  (MALDI-TOF-TOF)* | Phosphoglycerate kinase, [YP_695953] | 78 |
|  |  |  |  |  |  |  |
|  | Phosphoglycerate kinase *Clostridium difficile* 630, [Q181T8_CLODI](http://www.matrixscience.com/cgi/protein_view.pl?file=../data/20070313/FoTpOaSt.dat&hit=2) | K.ELASLAEIFVNDAFGTAHR.A | 115 | SP-5-MS-MS-2061  (MALDI-TOF-TOF)* | Phosphoglycerate kinase, [YP_695953] | 78 |
|  |  |  |  |  |  |  |
|  | Phosphoglycerate kinase *Clostridium difficile* 630, [Q181T8_CLODI](http://www.matrixscience.com/cgi/protein_view.pl?file=../data/20070313/FoTpOaSt.dat&hit=2) | K.FLGEAVANPVRPFTAILGGAK.V | 100 | SP-5-MS-MS-2128  (MALDI-TOF-TOF)* | Phosphoglycerate kinase, [YP_695953] | 78 |
|  |  |  |  |  |  |  |
| SP6 | Phosphoglycerate kinase *Clostridium difficile* 630, [Q181T8_CLODI](http://www.matrixscience.com/cgi/protein_view.pl?file=../data/20070313/FoTpOaSt.dat&hit=2) | K.ELASLAEIFVNDAFGTAHR.A  K.FLGEAVANPVRPFTAILGGAK.V | 167 | SP-6-MS-MS-COMBINED  (MALDI-TOF-TOF)* | Phosphoglycerate kinase, [YP_695953] | 78 |
|  |  |  |  |  |  |  |
|  | Phosphoglycerate kinase *Clostridium difficile* 630, [Q181T8_CLODI](http://www.matrixscience.com/cgi/protein_view.pl?file=../data/20070313/FoTpOaSt.dat&hit=2) | K.ELASLAEIFVNDAFGTAHR.A | 103 | SP-6-MS-MS-2060  (MALDI-TOF-TOF)* | Phosphoglycerate kinase, [YP_695953] | 78 |
|  |  |  |  |  |  |  |
|  | Phosphoglycerate kinase *Clostridium difficile* 630, [Q181T8_CLODI](http://www.matrixscience.com/cgi/protein_view.pl?file=../data/20070313/FoTpOaSt.dat&hit=2) | K.FLGEAVANPVRPFTAILGGAK.V | 101 | SP-6-MS-MS-2128  (MALDI-TOF-TOF)* | Phosphoglycerate kinase, [YP_695953] | 78 |
|  |  |  |  |  |  |  |
| SP7 | Putative surface/cell-adhesion protein, multiple big2 domain.- *Clostridium tetani*, [Q897I8_CLOTE](http://hpdatastation/mascot/cgi/protein_view.pl?file=../data/20070222/F014753.dat&hit=Q897I8_CLOTE&px=1&protscore=49.4979040458398&_mudpit=1000) | R.EFTTGKEK.K  K.LEPKPEEPK.E  K.DINLNLLGIK.R  K.DINLNLLGIK.R  K.VHDEGNGKLSFGK.I | 49 | MS/MS  (ESI- TRAP) | Cell wall-associated serine proteinase, [YP_695569] | 25 |
|  |  |  |  |  |  |  |
| SP8 | Acetate kinase*.- Clostridium phytofermentans* ISDg, [Q1FFA7_9CLOT](http://www.matrixscience.com/cgi/protein_view.pl?file=../data/20070317/FoTrfice.dat&hit=Q1FFA7_9CLOT&px=1&_server_mudpit_switch=0.001) | K.VLVINCGSSSLK.Y | 50 | MS/MS  (ESI- TRAP) | Acetate kinase, [YP_694671] | 57 |
|  |  |  |  |  |  |  |
| SP9 | Aminopeptidase BH2245 [imported] - *Bacillus halodurans* (strain C-125), [E83930](http://www.matrixscience.com/cgi/protein_view.pl?file=../data/20070317/FoTrrrES.dat&hit=E83930&px=1&_server_mudpit_switch=0.001) | K.VGVNIQPGQTLVIR.T  K.VGVNIQPGQTLVIR.T  K.VGVNIQPGQTLVIR.T | 58 | MS/MS  (ESI- TRAP) | Aminopeptidase, [YP_696676] | 51 |
|  |  |  |  |  |  |  |
|  | Aminopeptidase BH2245 [imported] *- Bacillus halodurans* (strain C-125), [E83930](http://www.matrixscience.com/cgi/protein_view.pl?file=../data/20070313/FoTurnee.dat&hit=1) | K.VGVNIQPGQTLVIR.T | 87 | SP-9-MS-MS-1493  (MALDI-TOF-TOF)* | Aminopeptidase, [YP_696676] | 51 |
|  |  |  |  |  |  |  |
| SP10 | Aminopeptidase BH2245 [imported] - *Bacillus halodurans* (strain C-125), [E83930](http://www.matrixscience.com/cgi/protein_view.pl?file=../data/20070316/FoTeSiaT.dat&hit=E83930&px=1&_server_mudpit_switch=0.001) | K.VGVNIQPGQTLVIR.T  K.VGVNIQPGQTLVIR.T |  | MS/MS  (ESI- TRAP) | Aminopeptidase, [YP_696676] | 51 |
|  |  |  |  |  |  |  |
| SP11 | Methionine gamma-lyase -*Clostridium novyi* NT, [gi|118443737](http://www.matrixscience.com/cgi/protein_view.pl?file=../data/20070314/FoTuIGsw.dat&hit=gi|118443737&px=1&_server_mudpit_switch=0.001) | R.LSVGLETVDDIIADLK.Q | 73 | MS/MS  (ESI- TRAP) | Cystathionine beta-lyase, [ABG82803] | 40 |
|  |  |  |  |  |  |  |
| SP12 | Methionine gamma-lyase- *Clostridium tetani* E88, [gi|28212109](http://www.matrixscience.com/cgi/protein_view.pl?file=../data/20070314/FoTuIauT.dat&hit=gi|28212109&px=1&_server_mudpit_switch=0.001) | K.VVYLETPANPNLK.V | 62 | MS/MS  (ESI- TRAP) | Cystathionine beta-lyase, [YP_694633] | 45 |
|  |  |  |  |  |  |  |
| SP13 | Cell wall hydrolase/autolysin precursor- *Clostridium thermocellum* ATCC 27405, [Q4CD81_CLOTM](http://www.matrixscience.com/cgi/protein_view.pl?file=../data/20070317/FoTrfiut.dat&hit=Q4CD81_CLOTM&px=1&_server_mudpit_switch=0.001) | K.LADDAFR.Q  K.DINLDISLKLGK.L | 54 | MS/MS  (ESI- TRAP) | N-acetylmuramoyl-L-alanine amidase, [YP_697056] | 32 |
|  |  |  |  |  |  |  |
| SP14 | Seryl-tRNA synthetase, class IIa - *Alkaliphilus metalliredigenes* QYMF, [Q3C2S0_9CLOT](http://www.matrixscience.com/cgi/protein_view.pl?file=../data/20070316/FoTeieYm.dat&hit=Q3C2S0_9CLOT&px=1&_server_mudpit_switch=0.001) | R.ASMIGTGQLPK.F  R.ICTGDLGFTAAFK.Y | 116 | MS/MS  (ESI- TRAP) | Seryl-tRNA synthetase, [YP_694487] | 66 |
|  |  |  |  |  |  |  |
| SP15 | Ornithine carbamoyltransferase- *Clostridium beijerincki* NCIMB 8052, [Q2WM43_CLOBE](http://www.matrixscience.com/cgi/protein_view.pl?file=../data/20070313/FoTpOecw.dat&hit=Q2WM43_CLOBE&px=1&_server_mudpit_switch=0.001) | R.MYDGIEYR.G  K.GVNFVYMGDAR.N | 92 | SP-15-MS-MS-COMBINED  (MALDI-TOF-TOF)* | Ornithine carbamoyltransferase, [YP_694626] | 60 |
|  |  |  |  |  |  |  |
|  | Ornithine carbamoyltransferase- *Clostridium beijerincki* NCIMB 8052, [Q2WM43_CLOBE](http://www.matrixscience.com/cgi/protein_view.pl?file=../data/20070313/FoTpObee.dat&hit=5) | R.MYDGIEYR.G  R.MYDGIEYR.G  K.GVNFVYMGDAR.N  K.GVNFVYMGDAR.N  K.QSVVFDEAENR.M  R.LKGVNFVYMGDAR.N  K.NADVIYTDVWVSMGEPDEVWESR.L | 41  (16.6%) | PMF | Ornithine carbamoyltransferase, [YP_694626] | 60 |
|  |  |  |  |  |  |  |
|  | Ornithine carbamoyltransferase- *Clostridium beijerincki* NCIMB 8052, [Q2WM43_CLOBE](http://www.matrixscience.com/cgi/protein_view.pl?file=../data/20070313/FoTpObTe.dat&hit=1) | R.MYDGIEYR.G | 45 | SP-15-MS-MS-1046  (MALDI-TOF-TOF)* | Ornithine carbamoyltransferase, [YP_694626] | 60 |
|  |  |  |  |  |  |  |
|  | Ornithine carbamoyltransferase- *Clostridium beijerincki* NCIMB 8052, [Q2WM43_CLOBE](http://www.matrixscience.com/cgi/protein_view.pl?file=../data/20070313/FoTpObeO.dat&hit=1) | K.GVNFVYMGDAR.N | 81 | SP-15-MS-MS-1228  (MALDI-TOF-TOF)* | Ornithine carbamoyltransferase, [YP_694626] | 60 |
|  |  |  |  |  |  |  |
| SP21 | Transketolase C-terminal section- *Clostridium* sp. OhILAs, [Q1ETF5_9CLOT](http://www.matrixscience.com/cgi/protein_view.pl?file=../data/20070319/FoTrlrsm.dat&hit=Q1ETF5_9CLOT&px=1&_server_mudpit_switch=0.001) | K.MEGPVYVR.L  K.MEGPVYVR.L  K.MEGPVYVR.L  R.TIPNMTVIVPADGVETK.A | 56 | MS/MS  (ESI- TRAP) | Putative transketolase, C-terminal subunit, [YP_694753] | 69 |
|  |  |  |  |  |  |  |
| SP24 | Putative deoxyribose-phosphate aldolase- *Clostridium difficile* 630, [Q18C22_CLODI](http://www.matrixscience.com/cgi/protein_view.pl?file=../data/20070316/FoTelbHm.dat&hit=Q18C22_CLODI&px=1&_server_mudpit_switch=0.001) | K.TSTGMGTGGATLEDIK.L  K.TSTGMGTGGATLEDIK.L  K.TSTGMGTGGATLEDIK.L  K.TSTGMGTGGATLEDIK.L  K.VIIETCYLDEDEK.I  K.VIIETCYLDEDEK.I  K.VIIETCYLDEDEK.I | 70 | MS/MS  (ESI- TRAP) | Deoxyribose-phosphate aldolase, [Q8XIR2] | 51 |
|  |  |  |  |  |  |  |
| SP25 | Translation elongation factor G, EF-G- *Bacillus stearothermophilus*, [Q9F4B2_BACST](http://www.matrixscience.com/cgi/protein_view.pl?file=../data/20070301/FogrSzaO.dat&hit=Q9F4B2_BACST&px=1&protscore=150.74&_mudpit=1000) | K.VEANVGAPQVAYR.E  R.EFKVEANVGAPQVAYR.E | 151 | SP-25-COMBINEDMS-MS  (MALDI-TOF-TOF) | Elongation factor G (EF-G), [Q8XHS1] | 67 |
|  |  |  |  |  |  |  |
|  | Translation elongation factor G:Small GTP-binding protein domain- *Exiguobacterium sibiricum* 255-15, [Q41A29_9BACI](http://www.matrixscience.com/cgi/protein_view.pl?file=../data/20070301/FogrSzHe.dat&hit=1) | R.EFKVEANVGAPQVAYR.E | 101 | SP-25-MS-MS-1777  (MALDI-TOF-TOF)* | Elongation factor G (EF-G), [Q8XHS1] | 65 |
|  |  |  |  |  |  |  |
|  | Translation elongation factor G- *Clostridium difficile* 630, [Q18CF4_CLODI](http://www.matrixscience.com/cgi/protein_view.pl?file=../data/20070301/FogrSzTw.dat&hit=1) | R.ATYTMIFDHYEQVPASVAK.K | 56 | SP-25-MS-MS-2171  (MALDI-TOF-TOF)* | Elongation factor G (EF-G), [Q8XHS1] | 66 |
|  |  |  |  |  |  |  |
|  | Elongation factor EF-2- *Bacillus* sp. NRRL B-14911, [Q2B2L1_9BACI](http://www.matrixscience.com/cgi/protein_view.pl?file=../data/20070301/FogrSzEw.dat&hit=1) | K.VEANVGAPQVAYR.E | 80 | SP-25-MS--MS-1373  (MALDI-TOF-TOF)* | Elongation factor G (EF-G), [Q8XHS1] | 66 |
|  |  |  |  |  |  |  |
| SP26 | Triosephosphate isomerase (Fragment)- *Clostridium bifermentans*, [Q64G22_CLOBI](http://www.matrixscience.com/cgi/protein_view.pl?file=../data/20070301/FogrSaYw.dat&hit=Q64G22_CLOBI&px=1&protscore=218.04&_mudpit=1000) | -.TIAEAVEFVNEIK.G  K.ELDIDYVVLGHSER.R  K.ALEAGIDPILCIGETLEER.E | 218 | SP-26-C0MBINED-MS-MS  (MALDI-TOF-TOF)* | Triosephosphate isomerase, [Q0TQY8] | 54 |
|  |  |  |  |  |  |  |
|  | Triosephosphate isomerase (Fragment)- *Clostridium bifermentans*, [Q64G22_CLOBI](http://www.matrixscience.com/cgi/protein_view.pl?file=../data/20070301/FogrSbct.dat&hit=1) | K.ALENVTAEYMK.K  -.TIAEAVEFVNEIK.G  R.QYFNETNETVNK.K  R.QYFNETNETVNKK.V  R.RQYFNETNETVNK.K  K.ELDIDYVVLGHSER.R  K.VNNTDVEAVICAPFTLLK.D  K.ALEAGIDPILCIGETLEER.E | 79  (60.1%) | PMF | Triosephosphate isomerase, [Q0TQY8] | 54 |
|  | Triosephosphate isomerase (Fragment)- *Clostridium bifermentans*, [Q64G22_CLOBI](http://www.matrixscience.com/cgi/protein_view.pl?file=../data/20070301/FogrSfEe.dat&hit=1) | TIAEAVEFVNEIK.G | 94 | SP-26-MS-MS-1462  (MALDI-TOF-TOF)* | Triosephosphate isomerase, [Q0TQY8] | 54 |
|  |  |  |  |  |  |  |
|  | Triosephosphate isomerase (Fragment)- *Clostridium bifermentans*, [Q64G22_CLOBI](http://www.matrixscience.com/cgi/protein_view.pl?file=../data/20070301/FogrSfHe.dat&hit=1) | K.ELDIDYVVLGHSER.R | 129 | SP-26-MS-MS-1644  (MALDI-TOF-TOF)* | Triosephosphate isomerase, [Q0TQY8] | 54 |
|  |  |  |  |  |  |  |
|  | Triosephosphate isomerase (Fragment)- *Clostridium bifermentans*, [Q64G22_CLOBI](http://www.matrixscience.com/cgi/protein_view.pl?file=../data/20070301/FogrSfut.dat&hit=1) | K.ALEAGIDPILCIGETLEER.E | 95 | SP-26-MS-MS-2099  (MALDI-TOF-TOF)* | Triosephosphate isomerase, [Q0TQY8] | 54 |
|  |  |  |  |  |  |  |
| SP27 | Elongation factor P- *Clostridium* sp. OhILAs, [Q1EX90_9CLOT](http://www.matrixscience.com/cgi/protein_view.pl?file=../data/20070317/FoTrfxcE.dat&hit=Q1EX90_9CLOT&px=1&_server_mudpit_switch=0.001) | R.EEAFNPSDKFPK.A | 36 | MS/MS  (ESI- TRAP) | Translation elongation factor P, [Q0TPC2] | 69 |
|  |  |  |  |  |  |  |
| SP28 | Elongation factor P- *Clostridium* sp. OhILAs, [Q1EX90_9CLOT](http://www.matrixscience.com/cgi/protein_view.pl?file=../data/20070316/FoTeSaHE.dat&hit=Q1EX90_9CLOT&px=1&_server_mudpit_switch=0.001) | R.EEAFNPSDKFPK.A | 38 | MS/MS  (ESI- TRAP) | Translation elongation factor P, [Q0TPC2] | 69 |
|  |  |  |  |  |  |  |
| SP44 | Cell surface protein- *Bacillus cereus* (strain ATCC 14579 / DSM 31), [Q816D8_BACCR](http://hpdatastation/mascot/cgi/protein_view.pl?file=../data/20070306/F014820.dat&hit=Q816D8_BACCR&px=1&protscore=47.8079040458398&_mudpit=1000) | K.EPQVK.T  K.EPQVK.T  K.EPQVK.T  K.VKDEVK.V  K.IPEDPKEPK.E K.IPEDPKEPK.E | 48 | MS/MS  (ESI- TRAP) | Rhomboid family protein, [YP_696141] | 29 |
|  |  |  |  |  |  |  |
| **B. Cell wall proteins** | | |  |  |  |  |
| MP-1 | Rubredoxin-type Fe(Cys)4 protein- *Desulfitobacterium hafniense* DCB-2  [Q41Y22_DESHA](http://www.matrixscience.com/cgi/protein_view.pl?file=../data/20060727/FLnurxcn.dat&hit=Q41Y22_DESHA&px=1&protscore=134.71&_mudpit=1000) | K.AFLGLLNR.Y  K.AFLGLLNR.Y  R.IAFEEAEHAAK.F  R.EGYPEVAEAYKR.I | 135 | MS/MS  (ESI-QUAD-TOF) | Rubredoxin, [Q0TT07 ], [ABG84689, ABG84965, ABG83958] | 64 |
| MP-2 | Rubrerythrin:Rubredoxin-type Fe(Cys)4 protein- *Desulfitobacterium hafniense* DCB-2.  [Q41Y22_DESHA](http://www.matrixscience.com/cgi/protein_view.pl?file=../data/20060727/FLnurxaT.dat&hit=Q41Y22_DESHA&px=1&protscore=135.25&_mudpit=1000) | K.AFLGLLNR.Y  K.AFLGLLNR.Y  K.AFLGLLNR.Y  R.IAFEEAEHAAK.F  R.EGYPEVAEAYKR.I | 135 | MS/MS  (ESI-QUAD-TOF) | Rubredoxin/rubrerythrin, [Q0TM31], [ABG84689, ABG84965, ABG83958] | 74 |
| MP-3 | Rubrerythrin:Rubredoxin-type Fe(Cys)4 protein- *Desulfitobacterium hafniense* DCB-2.  [Q41Y22_DESHA](http://www.matrixscience.com/cgi/protein_view.pl?file=../data/20060727/FLnurxue.dat&hit=Q41Y22_DESHA&px=1&protscore=214.31&_mudpit=1000) | K.AFLGLLNR.Y  K.AFLGLLNR.Y  K.AFLGLLNR.Y  R.IAFEEAEHAAK.F  R.HGKAFLGLLNR.Y  K.RIAFEEAEHAAK.F  R.EGYPEVAEAYKR.I | 214 | MS/MS  (ESI-QUAD-TOF) | Rubredoxin/rubrerythrin, [Q0TM31], [ABG84689, ABG84965, ABG83958] | 74 |
| MP-4 | Protein-synthesizing GTPase (Translation elongation factor Tu (EF-TU)) – *B. thuringiensis*. | R.GTVATGRVER.G  R.QVGVPYIVVFLNK.C  K.LLDQAQAGDNIGALLR.G  R.KLLDQAQAGDNIGALLR.G | 290 | MS/MS  (ESI-QUAD-TOF) | Translation elongation factor Tu, [Q0TMN0], [YP_697096, YP_697110] | 70 |
| MP-5 | H(+)-transporting ATP synthase beta chain- *Oceanobacillus iheyensis*.  [Q8EM83_OCEIH](http://www.matrixscience.com/cgi/protein_view.pl?file=../data/20060727/FLnurxsE.dat&hit=Q8EM83_OCEIH&px=1&protscore=373.943305028184&_mudpit=1000) | K.IGLFGGAGVGK.T  K.VVDLLAPYIK.G  K.VVDLLAPYIKGGK.I  R.FTQAGSEVSALLGR.M  R.VALTGLTMAEYFR.D  R.DEQGQDVLLFVDNIFR.F | 374 | MS/MS  (ESI-QUAD-TOF) | ATP synthase F1, beta subunit, [Q0TNC4], [YP_696866] | 67 |
| MP-6 | ATP synthase beta chain- *Mycoplasma synoviae*.  [Q4A604_MYCS5](http://www.matrixscience.com/cgi/protein_view.pl?file=../data/20060727/FLnurxsT.dat&hit=Q4A604_MYCS5&px=1&protscore=304.46&_mudpit=1000) | K.IGLFGGAGVGK.T  R.FTQAGSEVSALLGR.I  R.VALTGLTMAEYFR.D  R.VALTGLTMAEYFR.D  K.TALVFGQMNEPPGAR.M  K.TALVFGQMNEPPGAR.M | 304 | MS/MS  (ESI-QUAD-TOF) | ATP synthase F1, beta subunit, [Q0TNC4],  [YP_696866] | 63 |
| MP-7 | H(+)-transporting ATP synthase beta chain- *Oceanobacillus iheyensis*.  [Q8EM83_OCEIH](http://www.matrixscience.com/cgi/protein_view.pl?file=../data/20060727/FLnurzun.dat&hit=Q8EM83_OCEIH&px=1&protscore=383.643305028184&_mudpit=1000) | K.IGLFGGAGVGK.T  K.VVDLLAPYIK.G  K.VVDLLAPYIKGGK.I  R.FTQAGSEVSALLGR.M  R.VALTGLTMAEYFR.D  R.DEQGQDVLLFVDNIFR.F | 384 | MS/MS  (ESI-QUAD-TOF) | ATP synthase F1, beta subunit, [Q0TNC4],  [YP_696866] | 67 |
| MP-8 | H+-transporting two-sector ATPase alpha chain – *B. megaterium.*  [F31482](http://www.matrixscience.com/cgi/protein_view.pl?file=../data/20060727/FLnurzse.dat&hit=F31482&px=1&protscore=169.76&_mudpit=1000) | K.HVLVIYDDLTK.Q  R.KSVHEPLQTGIK.A  R.EAYPGDVFYLHSR.L | 170 | MS/MS  (ESI-QUAD-TOF) | ATP synthase F1, alpha subunit, [Q0TNC2],  [YP_696868] | 65 |
| MP-9 | H+-transporting two-sector ATPase alpha chain – *B. megaterium.*  [F31482](http://www.matrixscience.com/cgi/protein_view.pl?file=../data/20060727/FLnurzsO.dat&hit=F31482&px=1&protscore=196.64&_mudpit=1000) | K.SVHEPLQTGIK.A  K.HVLVIYDDLTK.Q  R.KSVHEPLQTGIK.A  R.EAYPGDVFYLHSR.L | 197 | MS/MS  (ESI-QUAD-TOF) | ATP synthase F1, alpha subunit, [Q0TNC2],  [YP_696868] | 65 |
| MP-10 | H+-transporting two-sector ATPase alpha chain – *B. megaterium.*  [F31482](http://www.matrixscience.com/cgi/protein_view.pl?file=../data/20060727/FLnurzee.dat&hit=F31482&px=1&protscore=175.22&_mudpit=1000) | K.SVHEPLQTGIK.A  K.HVLVIYDDLTK.Q  R.KSVHEPLQTGIK.A  R.EAYPGDVFYLHSR.L | 175 | MS/MS  (ESI-QUAD-TOF) | ATP synthase F1, alpha subunit, [Q0TNC2],  [YP_696868] | 65 |
|  |  |  |  |  |  |  |
| **C. Proteins differentially expressed on cooked meat medium** | | |  |  |  |  |
|  |  |  |  |  |  |  |
| CMM-2 | 2-hydroxyacyl-CoA dehydratase (Fragment), *Clostridium difficile*, [Q5U923_CLODI](http://hpdatastation/mascot/cgi/protein_view.pl?file=../data/20070605/F015607.dat&hit=Q5U923_CLODI&px=1&protscore=79.0438177421602&_mudpit=1000) | K.SGFFMR.K  K.SGFFMR.K  K.IHESIEVYNEHR.K  K.IHESIEVYNEHR.K | 79 | MS/MS  (ESI-TRAP) | Riboflavin biosynthesis protein, [YP_695002] | 22 |
|  |  |  |  |  |  |  |
| CMM-3 | Ornithine carbamoyltransferase, *Clostridium beijerincki*, [Q2WM43_CLOBE](http://hpdatastation/mascot/cgi/protein_view.pl?file=../data/20070605/F015595.dat&hit=Q2WM43_CLOBE&px=1&protscore=208.29690887108&_mudpit=1000) | K.ESIADTAR.V  K.KESIADTAR.V  K.KESIADTAR.V  R.MYDGIEYR.G  R.NNMGNSLMVACAK.M  R.NNMGNSLMVACAK.M  R.NNMGNSLMVACAK.M | 208 | MS/MS  (ESI-TRAP) | Ornithine carbamoyltransferase, catabolic, [YP_694626] | 60 |
|  |  |  |  |  |  |  |
| CMM-4 | O-acetylhomoserine/O-acetylserine sulfhydrylase, *Clostridium beijerincki*, [Q2WQ73_CLOBE](http://hpdatastation/mascot/cgi/protein_view.pl?file=../data/20070605/F015608.dat&hit=Q2WQ73_CLOBE&px=1&protscore=46.9&_mudpit=1000) | R.AGDHIVSAK.T | 47 | MS/MS  (ESI-TRAP) | Cystathionine beta-lyase, [YP_694633] | 31 |
|  |  |  |  |  |  |  |
| CMM-5 | Threonine dehydratase catabolic, *Clostridium difficile*, [Q182J9_CLODI](http://hpdatastation/mascot/cgi/protein_view.pl?file=../data/20070601/F015560.dat&hit=Q182J9_CLODI&px=1&protscore=80.02&_mudpit=1000) | K.AVEIQK.E  K.ANGVIASSAGNHAQGVALGAK.M | 80 | MS/MS  (ESI-TRAP) | Threonine dehydratase, catabolic, [YP_695814] | 43 |
|  |  |  |  |  |  |  |
| CMM-6 | Threonine dehydratase catabolic, *Clostridium difficile*, [Q182J9_CLODI](http://hpdatastation/mascot/cgi/protein_view.pl?file=../data/20070622/F015770.dat&hit=Q182J9_CLODI&px=1&protscore=217.96&_mudpit=1000) | K.AVEIQK.E  K.ALNPNVK.I  K.ALNPNVK.I  K.CENLQK.T  K.CENLQK.T  K.IASLTDEEK.A  K.IASLTDEEK.A  K.ETGATFLHPFNDK.Y  K.ETGATFLHPFNDK.Y  K.ANGVIASSAGNHAQGVALGAK.M  K.ANGVIASSAGNHAQGVALGAK.M | 218 | MS/MS  (ESI-TRAP) | Threonine dehydratase, catabolic, [YP_695814] | 43 |
|  |  |  |  |  |  |  |
| CMM-7 | Acyl-CoA dehydrogenase, short-chain specific, *Clostridium difficile*, [Q188I5_CLODI](http://hpdatastation/mascot/cgi/protein_view.pl?file=../data/20070618/F015699.dat&hit=Q188I5_CLODI&px=1&protscore=249.03790404584&_mudpit=1000) | K.ENLLGK.E  K.ENLLGK.E  R.GTSAFIVESK.W  K.ALQLHGGYGFIK.D  R.GTETSDLIFENVK.V  K.FQNTQFTIADMETK.V  K.FQNTQFTIADMETK.V  K.FQNTQFTIADMETK.V  K.FQNTQFTIADMETK.V  K.FQNTQFTIADMETK.V  K.FQNTQFTIADMETK.V | 249 | MS/MS  (ESI-TRAP) | Butyryl-CoA dehydrogenase, [YP_696974] | 49 |
|  |  |  |  |  |  |  |
| CMM-8 | Acyl-CoA dehydrogenase, short-chain specific, *Clostridium difficile*, [Q188I5_CLODI](http://hpdatastation/mascot/cgi/protein_view.pl?file=../data/20070605/F015597.dat&hit=Q188I5_CLODI&px=1&protscore=250.43790404584&_mudpit=1000) | K.ENLLGK.E  R.GTSAFIVESK.W  R.GTSAFIVESK.W  K.ALQLHGGYGFIK.D  R.GTETSDLIFENVK.V  K.FQNTQFTIADMETK.V  K.FQNTQFTIADMETK.V | 250 | MS/MS  (ESI-TRAP) | Butyryl-CoA dehydrogenase, [YP_696974] | 49 |
|  |  |  |  |  |  |  |
| CMM-9 | Butyryl-CoA dehydrogenase, [imported], *Clostridium thermosaccharolyticum*, [T45286](http://hpdatastation/mascot/cgi/protein_view.pl?file=../data/20070611/F015637.dat&hit=T45286&px=1&protscore=74.8&_mudpit=1000) | K.ENLLGK.E  K.IAMATLDGGRIGIAAQR.L | 75 | MS/MS  (ESI-TRAP) | Butyryl-CoA dehydrogenase, [YP_696974] | 68 |
|  |  |  |  |  |  |  |
| CMM-10 | 2-hydroxyacyl-CoA dehydratase (Fragment), *Clostridium difficile*, [Q5U923_CLODI](http://hpdatastation/mascot/cgi/protein_view.pl?file=../data/20070601/F015559.dat&hit=Q5U923_CLODI&px=1&protscore=80.5138177421602&_mudpit=1000) | K.SGFFMR.K  K.SGFFMR.K  K.IHESIEVYNEHR.K  K.IHESIEVYNEHR.K | 81 | MS/MS  (ESI-TRAP) | Riboflavin biosynthesis protein RibA, [YP_695002] | 22 |
|  |  |  |  |  |  |  |
| CMM-11 | Peptide synthetase, *Bacillus thuringiensis serovar* israelensis, [Q3ESC5_BACTI](http://hpdatastation/mascot/cgi/protein_view.pl?file=../data/20070605/F015603.dat&hit=Q3ESC5_BACTI&px=1&protscore=66.8469088710801&_mudpit=1000) | K.ILLDGR.T  R.KILLDGR.T | 67 | MS/MS  (ESI-TRAP) | UDP-glucose 4-epimerase, [YP_694941] | 23 |
|  |  |  |  |  |  |  |
| CMM-12 | Electron transfer flavoprotein beta-subunit- *Clostridium difficile*, Q188I4_CLODI | K.IIIGLK.Q  K.ETGTLIR.D  K.QVPDTNEVR.I  R.INKETGTLIR.D  R.QAIDGDTAQVGPQIAEK.L  R.QAIDGDTAQVGPQIAEK.L | 177 | MS/MS  (ESI-TRAP) | Electron transfer flavoprotein, beta subunit, [YP_696973] | 60 |

**^a^** Homologous protein in *C. perfringens* ATCC13124 proteome was searched using SWISS-PROT data base at http://www.expasy.org/uniprot.

**^b^** Protein accession number and name are shown.

**^c^**Values in parenthesis indicate % coverage of PMF result.

*****MS/MS data of selected peptide was used for database search.
