# Supplementary figures and images for: Differential proteomic analysis of Clostridium perfringens ATCC13124; identification of dominant, surface and structure associated proteins
Source: BMC Microbiol. 2009 Aug 10;9:162. doi: 10.1186/1471-2180-9-162 (PMC2731776; doi:10.1186/1471-2180-9-162)

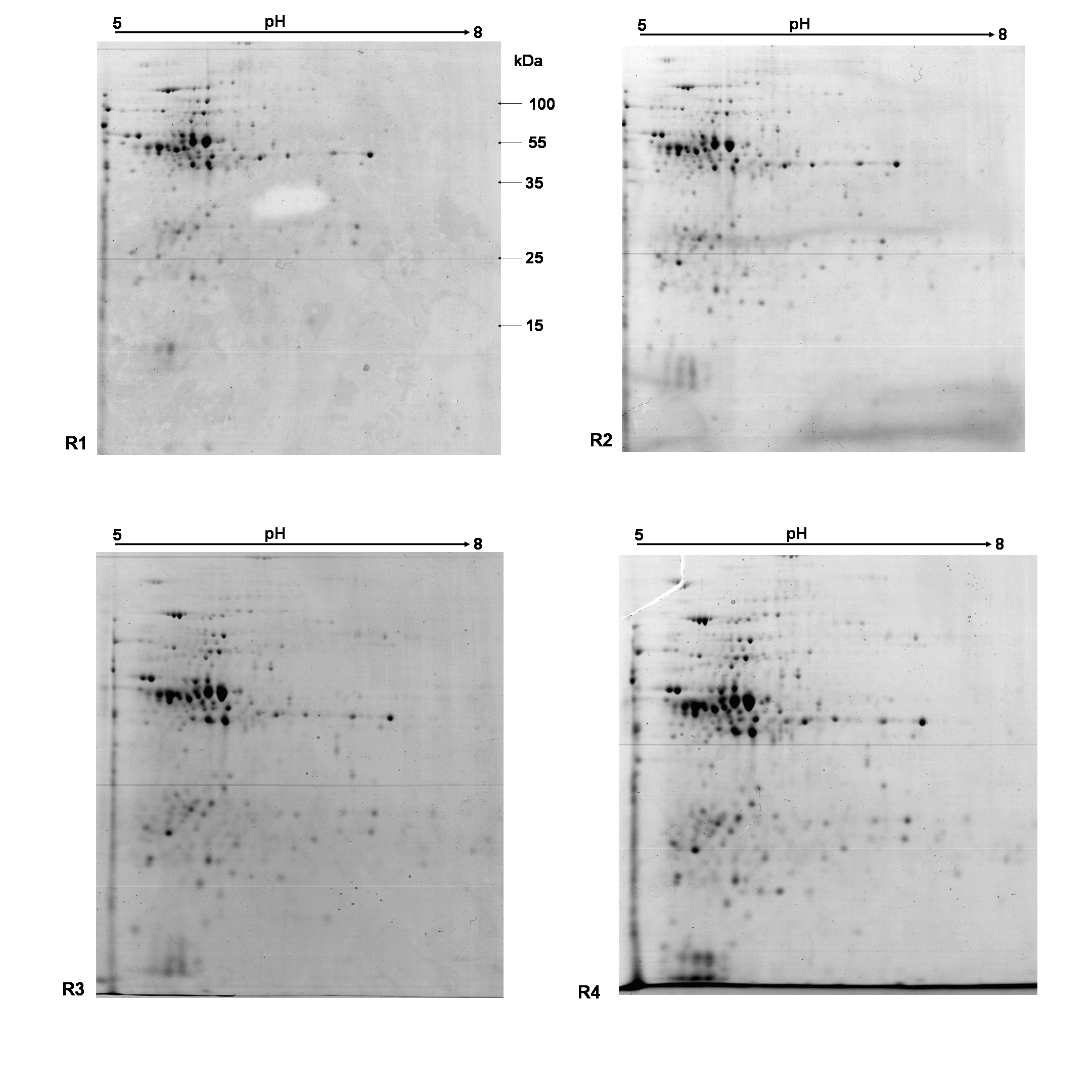

Supplement: Additional file 3 — Whole cell proteome of Clostridium perfringens ATCC13124 grown on cooked meat medium. Proteins were separated by 2-DE. Approximately 500 μg of total cellular proteins were separated on 17 cm IPG strips (pH 5–8) and stained with Coomassie brilliant blue R250. R1 and R2 are analytical replicates of experiment-1 while R3 and R4 are analytical replicates of experiment 2. [file 1471-2180-9-162-S3.tiff]

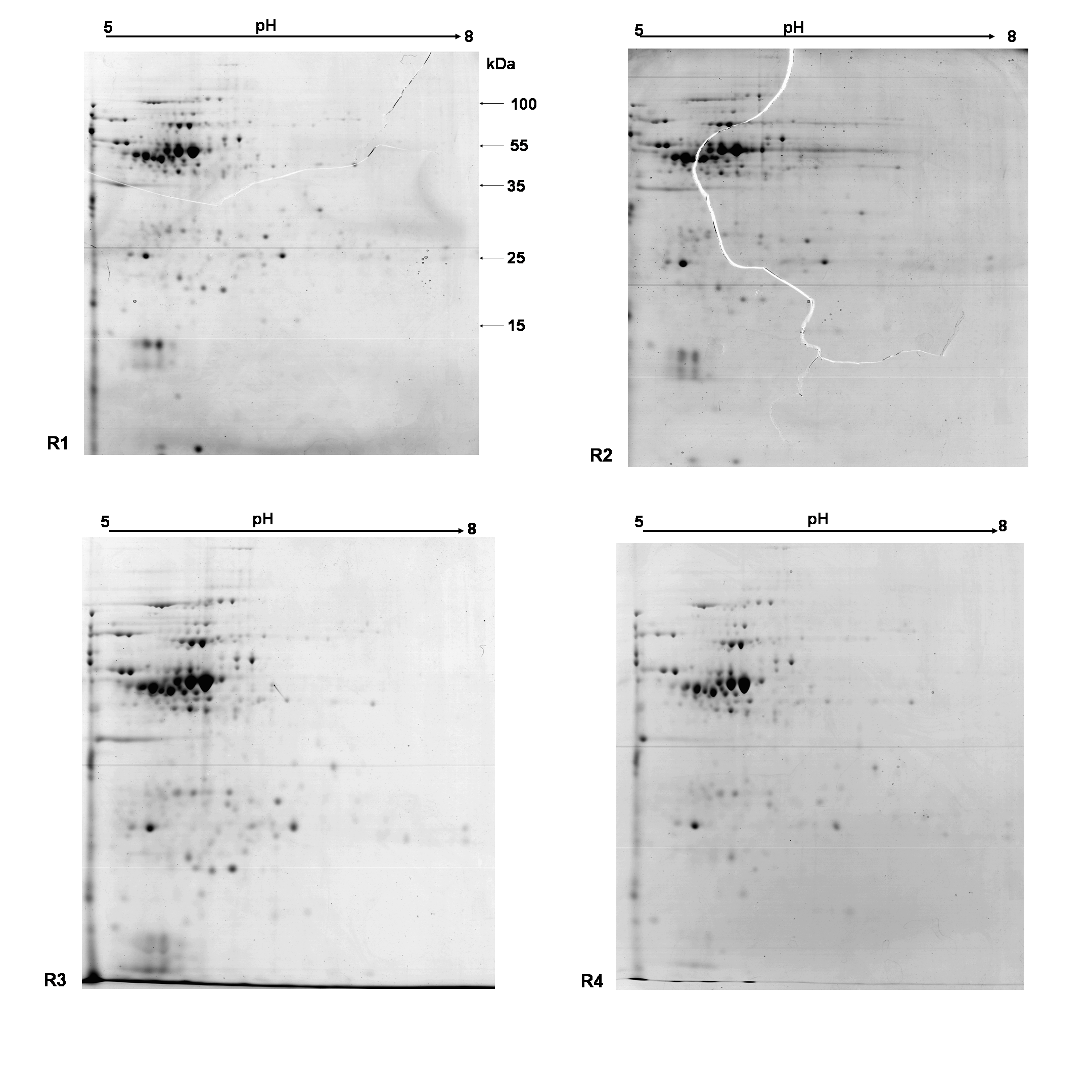

Supplement: Additional file 4 — Whole cell proteome of Clostridium perfringens ATCC13124 grown on TPYG medium. Proteins were separated by 2-DE. Approximately 500 μg of total cellular proteins were separated on 17 cm IPG strips (pH 5–8) and stained with Coomassie brilliant blue R250. R1 and R2 are analytical replicates of experiment-1 while R3 and R4 are analytical replicates of experiment 2. [file 1471-2180-9-162-S4.tiff]

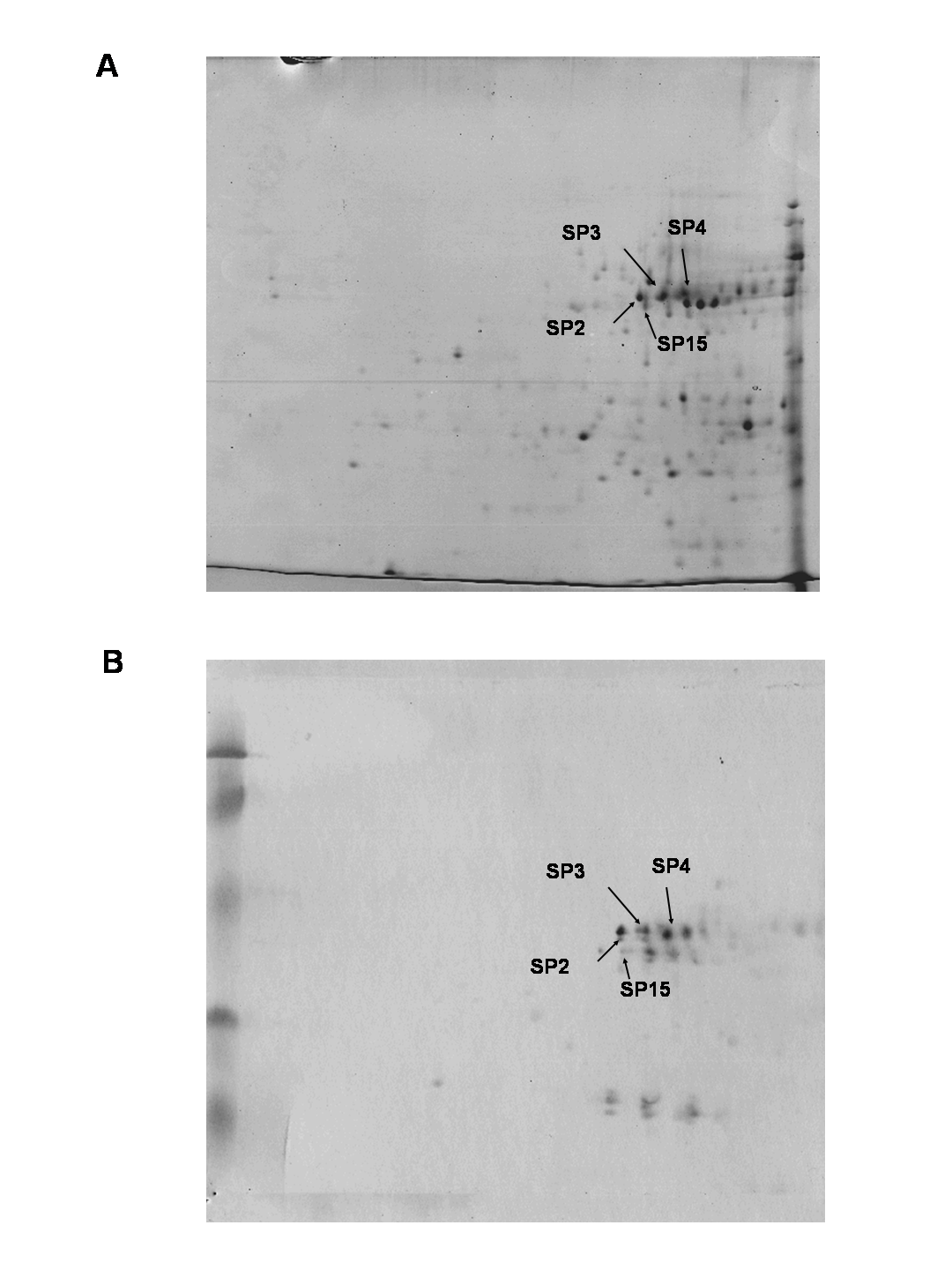

Supplement: Additional file 5 — Western blot analysis of immunogenic surface proteins from C. perfringens ATCC13124. Surface protein fraction was separated by 2-DE and probed with mouse anti- C. perfringens (heat killed whole cell) serum. Goat anti-mouse HRP conjugate was used as secondary antibody (1:2000 dilutions) and blots were developed using Immuno-Blot HRP assay kit (Bio-Rad, USA) as per manufacturer's instructions. A, Coomassie stained 2-DE gel; B, corresponding blot as described above. Spots identified in this study are indicated with arrows. [file 1471-2180-9-162-S5.tiff]
