# Supplementary material for: Differential proteomic analysis of Clostridium perfringens ATCC13124; identification of dominant, surface and structure associated proteins
Source: BMC Microbiol. 2009 Aug 10;9:162. doi: 10.1186/1471-2180-9-162 (PMC2731776; doi:10.1186/1471-2180-9-162)
Supplement: Additional file 6 — Proteins identified in this study and their homologues in other bacteria. A few pathogenic organisms where the presence of respective protein has been shown experimentally in other studies are listed along with their localization and predicted role. [file 1471-2180-9-162-S6.doc]

**Additional file 6:** Proteins identified in this study and their homologues in other bacteria. A few pathogenic organisms where the presence of respective protein has been shown experimentally in other studies are listed along with their localization and predicted role.

| ***C. perfringens* protein** | **Homologue in**  **(No. of strain)** | **Amino acid identity (%)** | **Amino acid similarity (%)** | **Experimentally shown in** | **Reference** |
| --- | --- | --- | --- | --- | --- |
|  |  |  |  |  |  |
| **Translation, ribosomal structure and biogenesis (J)** | |  |  |  |  |
| Seryl-tRNA synthetase **(SP)** | *C. perfringens* (9)  *C. botulinum* (9)  *C. novyi* (1)  *C. tetani* (1)  *C. kluyveri* (1)  *Eubacterium ventriosum* (1) | 100  74-84  78  75  74  69 | 100  88-93  90  89  89  84 | *Bacillus anthracis* **(EC)**  *Streptococcus pyogenes* **(CE)** | **[5]**  **[6]** |
| Elongation factor G (EF-G) **(SP)** | *C. perfringens* (8)  *C. botulinum* (10)  *C. beijerinckii* (1)  *C. butyricum* (1)  *C. novyi* (1)  *C. tetani* (1)  *Thermoanaerobacter tengcongensis* (1) | 100  75-85  82  82  80  78  71 | 100  89-93  92  92  89  89  85 | *Bacillus anthracis* **(Sp, I, EC)**  *C. perfringens* **(WC, EC, I, V)**  *Streptococcus pyogenes* **(CE)**  *Streptococcus suis* **(EC)**  *Mycobacterium tuberculosis* **(EC)** | **[3, 5]**  **[2, 10]**  **[6]**  **[9]**  **[10]** |
| Translation elongation factor Tu **(CE)** | *C. perfringens* (8)  *C. botulinum* (9)  *C. butyricum* (1)  *C. beijerinckii* (1)  *C. tetani* (1)  *C. kluyveri* (1)  *C. novyi* (1)  *Thermoanaerobacter tengcongensis* (1) | 100  89-94  92  92  88  88  86  82 | 100  95-98  97  97  94  94  96  88 | *Helicobacter pylori* **(WC)**  *Bacillus anthracis* **(Sp, I, EC)**  *Streptococcus pyogenes* **(CE)**  *Streptococcus suis* **(EC)**  *Mycobacterium tuberculosis* **(EC)**  *Staphylococcus aureus* **(EC)**  *Chlamydia pneumoniae* **(WC, Vrl)**  *Coxiella burnetii* **(WC, Vrl)** | **[1]**  **[3, 5]**  **[6]**  **[9]**  **[10]**  **[14]**  **[15]**  **[17]** |
| Translation elongation factor P **(SP)** | *C. perfringens* (8)  *C. novyi* (1)  *C. botulinum* (10)  *C. butyricum* (1)  *C. kluyveri* (1)  *C. tetani* (1)  *Alkaliphilus oremlandii* (1) | 99-100  84  78-84  83  80  80  69 | 99-100  90  87-90  88  88  88  80 |  |  |
| **Cell envelope biogenesis, outer membrane (M)** | |  |  |  |  |
| N-acetylmuramoyl-L-alanine amidase **(SP)** | *C. perfringens* (8)  *C. tetani* (1)  *C. botulinum* (8)  *C. butyricum* (1)  *C. novyi* (1)  *C. kluyveri* (1)  *Alkaliphilus metalliredigens* (1) | 98-100  53  53-54  52  53  51  48 | 99-100  71  69-71  72  67  70  60 | *Staphylococcus aureus* **(EC)**  *Chlamydia pneumoniae* **(WC, Vrl)**  *Yersinia pestis* **(WC, Vrl)** | **[14]**  **[15]**  **[16]** |
| Choloylglycine hydrolase family protein **(SP)** | *C. perfringens* (8)  *Pediococcus pentosaceus* (1)  *Lactobacillus casei* (1) | 94-100  38  36 | 98-100  59  57 |  |  |
| UDP-glucose 4-epimerase **(CMM)** | *C. perfringens* (8)  *C. bolteae* (1)  *Ruminococcus gnavus* (1)  *Pseudoalteromonas atlantica* (1) | 97-100  61  61  62 | 99-100  78  78  77 |  |  |
| **Posttranslational modification, protein turnover (O)** | |  |  |  |  |
| Cell wall-associated serine proteinase **(SP)** | *C. perfringens* (8)  *C. botulinum* (1)  *C. novyi* (1)  *Thermoanaerobacter tengcongensis* (1) | 100  47  47  45 | 100  63  58  61 |  |  |
| **Energy production and conversion (C)** | |  |  |  |  |
| Acetate kinase **(SP)** | *C. perfringens* (8)  *C. botulinum* (4)  *C. beijerinckii* (1)  *Thermoanaerobacter pseudethanolicus* (1) | 99-100  62  61  62 | 99-100  79  81  79 |  |  |
| Rubredoxin/rubrerythrin **(CE)** | *C. perfringens* (8)  *C. novyi* (1)  *C. pasteurianum* (1)  *C. bolteae* (1)  *C. butyricum* (1)  *Ruminococcus gnavus* (1) | 98-100  81  77  73  73  71 | 98-100  90  83  83  84  84 |  |  |
| ATP synthase F1, alpha and beta subunit **(CE)** | *C. perfringens* (8)  *C. novyi* (1)  *C. botulinum* (10)  *C. butyricum* (1)  *C. beijerinckii* (1)  *C. paradoxum* (1)  *Alkaliphilus oremlandii* (1) | 99-100  82  81-82  82  82  77  76 | 99-100  93  92-93  92  92  89  89 | *Bacillus anthracis* **(Sp, EC)**  *Mycobacterium leprae* **(CE)**  *Mycobacterium bovis* **(CE)**  *Bacillus anthracis* **(Sp, I, EC)**  *Staphylococcus aureus* **(EC)** | **[3, 5]**  **[12]**  **[13]**  **[3, 5]**  **[14]** |
| Electron transfer flavoprotein, beta subunit **(CE)** | *C. perfringens* (8)  *C. botulinum* (2)  *C. beijerinckii* (1)  *C. butyricum* (1)  *C. novyi* (1)  *C. tetani* (1)  *Alkaliphilus metalliredigens* (1) | 99-100  71-80  78  77  77  70  66 | 99-100  83-90  89  88  86  86  81 | *Bacillus anthracis* **(EC)**  *Mycobacterium tuberculosis* **(EC)**  *Mycobacterium leprae* **(C)** | **[5]**  **[10]**  **[12]** |
| **Amino acid transport and metabolism (E)** | |  |  |  |  |
| Glutamate dehydrogenase **(SP)** | *C. perfringens* (8)  *C. botulinum* (2)  *C. butyricum* (1)  *C. novyi* (1)  *C. bartlettii* (1)  *B. halodurans* (1)  *Listeria monocytogenes* (7)  *C. tetani* | 99-100  74 – 75  73  74  71  71  70  34 | 99-100  87 – 88  87  86  86  84  83  53 |  |  |
| Glutamate synthase (NADPH), homotetrameric **(SP)** | *C. perfringens* (8)  *C. butyricum* (1)  *C. botulinum* (2)  *C. acetobutylicum* (1)  *C. novyi* (1) | 98-100  78  76  76  76 | 99-100  88  88  87  88 |  |  |
| Aminopeptidase **(SP)** | *C. perfringens* (8)  *C. tetani* (1)  *C. botulinum* (7)  *C. difficile* (1)  *C. beijerinckii* (1)  *C. butyricum*  *Desulfitobacterium hafniense* (2) | 99-100  62  58-59  57  58  58  57-58 | 99-100  78  76  76  75  74  76-77 | *Streptococcus suis* **(EC)** | **[9]** |
| Cystathionine beta-lyase **(SP, CMM)** | *C. perfringens* (8)  *C. acetobutylicum* (1)  *C. botulinum* (8)  *C. kluyveri* (1)  *C. beijerinckii* (1)  *C. butyricum* (1)  *Helicobacter hepaticus* (1) | 97-100  69  67-69  66  63  62  61 | 99-100  84  82-84  82  79  78  76 | *Bacillus anthracis* **(EC)**  *Coxiella burnetii* **(WC, Vrl)** | **[5]**  **[17]** |
| Ornithine carbamoyltransferase **(SP, CMM)** | *C. perfringens* (6)  *C. spiroforme* (1)  *C. ramosum* (1)  *C. difficile* (1)  *C. botulinum* (7)  *Streptococcus agalactiae* (10)  *Staphylococcus haemolyticus* (1)  *Staphylococcus epidermidis* (1)  *Staphylococcus aureus* (2)  *Mycoplasma capricolum* (1)  *Mycoplasma mycoides* (1) | 100  76  73  64  62  61  62  61  61  61  60 | 100  87  85  79  75  77  76  76  76  77  76 | *Streptococcus agalactiae* **(SP, IP)**  *Streptococcus pyogenes* **(CE, I)** | **[4]**  **[6]** |
| Threonine dehydratase, catabolic **(CMM)** | *C. perfringens* (8)  *C. kluyveri* (1)  *Fusobacterium nucleatum* (3)  *Alkaliphilus metalliredigens* (1)  *C. ramosum* (1)  *C. spiroforme* (1) | 96-100  59  52  53  51  52 | 98-100  82  76  74  73  72 |  |  |
| **Nucleotide transport and metabolism (F)** | |  |  |  |  |
| Deoxyribose-phosphate aldolase **(SP)** | *C. perfringens* (8)  *Lysinibacillus sphaericus* (1)  *Bacillus cereus* (1)  *Streptococcus sanguinis* (73)  *Streptococcus gordonii* (1)  *Listeria monocytogenes* (3)  *Bacillus weihenstephanensis* (1) | 100  73  75  73  72  75  74 | 100  87  86  84  85  83  87 |  |  |
| **Carbohydrate transport and metabolism (G)** | |  |  |  |  |
| Sucrose-6-phosphate dehydrogenase **(SP)** | *C. perfringens* (8)  *C. botulinum* (2)  *C. butyricum* (1)  *Bacillus licheniformis* (1) | 98-100  54-55  54  45 | 100  73-75  75  64 |  |  |
| Phosphoglycerate kinase **(SP)** | *C. perfringens* (8)  *C. botulinum* (2)  *C. butyricum* (1)  *C. novyi* (1) | 100  89  88  86 | 100  96  93  92 | *Streptococcus agalactiae* **(SP, IP)**  *Streptococcus pyogenes* **(CE, I)**  *Streptococcus oralis* **(SP)**  *Streptococcus suis* **(EC)**  *S. pneumoniae* **(SP, IP)**  *Bacillus anthracis* **(EC)**  *Chlamydia pneumoniae* **(WC)**  *Yersinia pestis* **(WC, Vrl)** | **[4]**  **[6]**  **[7]**  **[9]**  **[8]**  **[5]**  **[15]**  **[16]** |
| Putative transketolase, C-terminal subunit **(SP)** | *C. perfringens* (8)  *C. botulinum* (10)  *C. butyricum* (1)  *C. novyi* (1)  *C. kluyveri* (1)  *Alkaliphilus oremlandii* (1)  *Heliobacterium modesticaldum* (1) | 100  78-81  80  78  75  74  70 | 100  89-91  89  89  86  85  81 | *Streptococcus pyogenes* **(CE, I)** | **[6]** |
| Triosephosphate isomerase **(SP)** | *C. perfringens* (8)  *C. butyricum* (1)  *C. septicum* (1)  *C. botulinum* (10)  *C. tetani* (1)  *C. novyi* (1)  *Thermoanaerobacter tengcongensis* (1) | 100  81  83  70-79  68  68  62 | 100  87  93  82-86  81  77  76 |  |  |
| **Coenzyme transport and metabolism (H)** | |  |  |  |  |
| Riboflavin biosynthesis protein **(CMM)** | *C. perfringens* (8)  *C. novyi* (1)  *C. botulinum* (8)  *C. tetani* (1)  *Desulfitobacterium hafniense* (1) | 97-100  68  65-67  67  64 | 97-100  84  81-83  81  80 |  |  |
| **Lipid metabolism (I)** |  |  |  |  |  |
| Butyryl-CoA dehydrogenase **(CMM)** | *C. perfringens* (8)  *C. botulinum* (2)  *C. butyricum* (1)  *C. beijerinckii* (2)  *C. tetani* (1)  *C. novyi* (1)  *Thermoanaerobacter tengcongensis* (1) | 100  87  87  85  80  79  74 | 100  95  93  86  89  87  85 |  |  |
| **General function prediction only (R)** | |  |  |  |  |
| Rhomboid family protein **(SP)** | *C. perfringens* (8)  *C. botulinum* (2)  *C. beijerinckii* (1)  *C. tetani* (1)  *C. butyricum* (1) | 99-100  45  45  42  40 | 99-100  63  64  61  59 |  |  |

Letters in bold indicate proteome fraction where the protein has been identified. **SP** = surface protein, **CE** = cell envelope protein, **CMM** = protein overexpressed on CMM, **WC** = whole cell proteome, **Sp** = spore protein, **I** = immunogenic, **V** = VirR / VirS regulated protein, **IP** = immunogenic and protective, **EC** = extracellular, **C** = cytoplasmic, **Vrl** = predicted role in virulence.

1. Carlsohn E, Nystro¨m J Bo¨lin I, Nilsson CL, Svennerholm AM: **HpaA Is Essential for *Helicobacter pylori* Colonization in Mice.** *Infect Immun* 2006, **74:**920–926.
2. Shimizu T, Shima K, Yoshino K, Yonezawa K, Shimizu T, Hayashi H: **Proteome and transcriptome analysis of the virulence genes regulated by the VirR/VirS system in *Clostridium perfringens*.** *J Bacteriol* 2002, **184:**2587–2594.
3. DelVecchio VG, Connolly JP, Alefantis TG, Walz A, Quan MA, Patra G, Ashton JM, Whittington JT, Chafin RD, Liang X, Grewal P, Khan AS, Mujer CV: **Proteomic profiling and identification of immunodominant spore antigens of *Bacillus anthracis*, *Bacillus cereus*, and *Bacillus thuringiensis*.** *Appl Env Microbiol* 2006, **72:**6355–6363.
4. Hughes MJG, Moore JC, Lane JD, Wilson R, Pribul PK, Younes ZN, Dobson RJ, Everest P, Reason AJ, Redfern JM, Greer FM, Paxton T, Panico M, Morris HR, Feldman RJ, Santangelo JD: **Identification of major outer surface proteins of *Streptococcus agalactiae*.** *Infect Immun* 2002, **70:**1254–1259.
5. Walz A, Mujer CV, Connolly JP, Alefantis T, Chafin R, Dake C, Whittington J, Kumar SP, Khan AS, DelVecchio VG: ***Bacillus anthracis* secretome time course under host-simulated conditions and identification of immunogenic proteins.** *Prot Sci* 2007, **5:**11.
6. Cole JN, Ramirez RD, Currie BJ, Cordwell SJ, Djordjevic SP, Walker MJ: **Surface Analyses and Immune Reactivities of Major Cell Wall-Associated Proteins of Group A *Streptococcus*.** *Infect Immun* 2005, **73:**3137–3146
7. Wilkins JC, Beighton D, Homer KA: **Effect of acidic pH on expression of surface-associated proteins of *Streptococcus oralis*.** *Appl Environ Microbiol* 2003, **69:**5290–5296.
8. Ling E, Feldman G, Portnoi M, Dagan R, Overweg K, Mulholland F, Chalifa-Caspi V, Wells J, Mizrachi-Nebenzahl Y: **Glycolytic enzymes associated with the cell surface of *Streptococcus pneumoniae* are antigenic in humans and elicit protective immune responses in the mouse.** *Clin Exp Immunol* 2004, **138:**290–298.
9. Jing HB, Yuan J, JieWang, Yuan Y, Zhu L, Liu ZK, Zheng YL, Wei KH, Zhang ZM, Geng HR, Duan Q, Feng SZ, Yang RF, Cao WC, Wang HL, Jiang YQ: **Proteome analysis of *Streptococcus suis* serotype 2.** *Proteomics* 2008, **8:**333–349.
10. Kulkarni RR, Parreira VR, Sharif S, Prescott JF: ***Clostridium perfringens* antigens recognized by broiler chickens immune to necrotic entritis.** *Clin Vac Immunol* 2006, **13:**1358-1362.
11. Mattow J, Schaible UE, Schmidt F, Hagens K, Siejak F, Brestrich G, Haeselbarth G, Christinaüller E, Jungblut PR, Kaufmann SHE: **Comparative proteome analysis of culture supernatant proteins from virulent *Mycobacterium tuberculosis* H37Rv and attenuated *M. bovis* BCG Copenhagen.** *Electrophoresis* 2003, **24**:3405–3420.
12. Marques MAM, Neves-Ferreira AGC, Xavier da Silveira EK, Valente RH, Chapeaurouge A, Perales J, da Silva Bernardes R, Dobos KM, Spencer JS, Brennan PJ, Pessolani MCV: **Deciphering the proteomic profile of *Mycobacterium leprae* cell envelope.** *Proteomics* 2008, **8:**2477–2491.
13. Malen H, Berven FS, Søfteland T, Arntzen MO, D’Santos CS, De Souza GA, Wiker HG: **Membrane and membrane-associated proteins in Triton X-114 extracts of *Mycobacterium bovis* BCG identified using a combination of gel-based and gel-free fractionation strategies.** *Proteomics* 2008, **8:**1859–1870.
14. Pocsfalvi G, Cacace G, Cuccurullo M, Serluca G, Sorrentino A, Schlosser G, Blaiotta G, Malorni A: **Proteomic analysis of exoproteins expressed by enterotoxigenic *Staphylococcus aureus* strains.** *Proteomics* 2008, **8:**2462–2476.
15. Molestina RE, Klein JB, Miller RD, Pierce WH, Ramirez JA, Summersgill J: **Proteomic analysis of differentially expressed *Chlamydia pneumoniae* genes during persistent infection of HEp-2 cells.** *Infect Immun* 2002, **70:**2976-2981.
16. Chromy BA, Choi MW, Murphy GA, Gonzales AD, Corzett CH, Chang BC, Fitch JP, McCutchen-Maloney SI: **Proteomic characterization of *Yersinia pestis* virulence.** *Infect Immun* 2005, **187:**8172–8180.
17. Coleman SA, Fischer ER, Cockrell DC, Voth DE, Howe D, Mead DJ, Samuel JE, Heinzen RA: **Proteome and antigen profiling of *Coxiella burnetii* developmental forms.** *Infect Immun* 2007, **75:**290–298.
