# Supplementary material for: Differential proteomic analysis of Clostridium perfringens ATCC13124; identification of dominant, surface and structure associated proteins
Source: BMC Microbiol. 2009 Aug 10;9:162. doi: 10.1186/1471-2180-9-162 (PMC2731776; doi:10.1186/1471-2180-9-162)
Supplement: Additional file 7 — Pattern/profile, post translational modifications and topology search results for identified proteins of Clostridium perfringens. Proteins identified from different fractions, indicating theoretical localization. All the analysis was carried out using ExPASy Proteomics tools at http://www.expasy.ch. [file 1471-2180-9-162-S7.doc]

**Additional file 7:** Pattern / profile, post translational modifications and topology search results for proteins of *Clostridium perfringens* identified form different fractions, indicating theoretical localization. All the analysis was carried out using ExPASy Proteomics tools at [http://www.expasy.ch](http://www.expasy.ch/).

| ***C. perfringens* protein** | **Protein spots (1)** | **PROSITE (2)** | **SignalP (3)** | **SecretomeP (4)** | **TMHMM (5)** | **LipoP (6)** | **PSORTb (7)** |
| --- | --- | --- | --- | --- | --- | --- | --- |
|  |  |  |  |  |  |  |  |
| **Translation, ribosomal structure and biogenesis (J)** | |  |  |  |  |  |  |
| Seryl-tRNA synthetase **(SP)** | SP14 | Aminoacyl-transfer RNA synthetases class-II family profile | No | No | 0 | **-** | Cytoplasmic |
| Elongation factor G (EF-G) **(SP)** | SP25 | GTP-binding elongation factors signature | No | No | 0 | **-** | Cytoplasmic |
| Translation elongation factor Tu **(CE)** | MP4 | GTP-binding elongation factors signature | No | No | 0 | **-** | Cytoplasmic |
| Translation elongation factor P **(SP)** | SP27, SP28 | Elongation factor P signature | No | No | 0 | **-** | Cytoplasmic |
| **Cell envelope biogenesis, outer membrane (M)** | |  |  |  |  |  |  |
| N-acetylmuramoyl-L-alanine amidase **(SP)** | SP13 | No hit | Yes | No | 0 | SpI | Multiple localization |
| Choloylglycine hydrolase family protein **(SP)** | SP2 | no hit | No | Yes  (0.731) | 0 | **-** | Unknown |
| UDP-glucose 4-epimerase **(CMM)** | CMM11 | No hit | No | No | 0 | **-** | Unknown |
| **Posttranslational modification, protein turnover (O)** | |  |  |  |  |  |  |
| Cell wall-associated serine proteinase **(SP)** | SP7 | Gram-positive cocci surface proteins LPxTG motif profile  Serine proteases; subtilase family; aspartic acid, histidine and serine active site | Yes | Yes  (0.893) | 2 | SpI | Cell wall |
| **Energy production and conversion (C)** | |  |  |  |  |  |  |
| Acetate kinase **(SP)** | SP8 | Acetate and butyrate kinases family signature 1 & 2 | No | No | 0 | **-** | Cytoplasmic |
| Rubredoxin/rubrerythrin **(CE)** | MP1, MP2, MP3 | Rubredoxin-like domain profile | No | Yes  (0.901) | 0 | **-** | Cytoplasmic |
| ATP synthase F1, alpha subunit **(CE)** | MP8, MP9, MP10 | ATP synthase alpha and beta subunits signature | No | No | 0 | - | Cytoplasmic |
| ATP synthase F1, beta subunit **(CE)** | MP5, MP6, MP7 | ATP synthase alpha and beta subunits signature | No | No | 0 | **-** | Cytoplasmic |
| Electron transfer flavoprotein, beta subunit **(CE)** | CMM12 | Electron transfer flavoprotein beta-subunit signature | No | No | 0 | **-** | Unknown |
| **Amino acid transport and metabolism (E)** | |  |  |  |  |  |  |
| Glutamate dehydrogenase **(SP)** | SP1 | Glu / Leu / Phe / Val dehydrogenases active site | No | No | 0 | **-** | Cytoplasmic |
| Glutamate synthase (NADPH), homotetrameric **(SP)** | SP3 | Ferredoxin-type iron-sulfur binding domain profile | No | No | 0 | **-** | Cytoplasmic |
| Aminopeptidase **(SP)** | SP9, SP10 | No hit | No | No | 0 | **-** | Cytoplasmic |
| Cystathionine beta-lyase **(SP, CMM)** | SP11, SP12, CMM4 | Cys/Met metabolism enzymes pyridoxal-phosphate attachment site | No | No | 0 | **-** | Cytoplasmic |
| Ornithine carbamoyltransferase **(SP, CMM)** | SP15, CMM3 | Aspartate and ornithine carbamoyltransferases signature | No | No | 0 | **-** | Cytoplasmic |
| Threonine dehydratase, catabolic **(CMM)** | CMM5, CMM6 | Serine/threonine dehydratases pyridoxal-phosphate attachment site | No | No | 0 | **-** | Cytoplasmic |
| **Nucleotide transport and metabolism (F)** | |  |  |  |  |  |  |
| Deoxyribose-phosphate aldolase **(SP)** | SP24 | No hit | No | No | 0 | **-** | Cytoplasmic |
| **Carbohydrate transport and metabolism (G)** | |  |  |  |  |  |  |
| Sucrose-6-phosphate dehydrogenase **(SP)** | SP4 | Glycosyl hydrolases family 32 active site | No | No | 0 | **-** | Cytoplasmic |
| Phosphoglycerate kinase **(SP)** | SP5, SP6 | Phosphoglycerate kinase signature | No | No | 0 | **-** | Cytoplasmic |
| Putative transketolase, C-terminal subunit **(SP)** | SP21 | Transketolase signature 2 | No | No | 0 | **-** | Cytoplasmic |
| Triosephosphate isomerase **(SP)** | SP26 | Triosephosphate isomerase active site | No | No | 0 | **-** | Cytoplasmic |
| **Coenzyme transport and metabolism (H)** | |  |  |  |  |  |  |
| Riboflavin biosynthesis protein **(CMM)** | CMM2, CMM10 | No hit | No | No | 0 | **-** | Cytoplasmic |
| **Lipid metabolism (I)** |  |  |  |  |  |  |  |
| Butyryl-CoA dehydrogenase **(CMM)** | CMM7, CMM8, CMM9 | Acyl-CoA dehydrogenases signature 1 & 2 | No | No | 0 | **-** | Cytoplasmic |
| **General function prediction only (R)** | |  |  |  |  |  |  |
| Rhomboid family protein **(SP)** | SP44 | No hit | No | Yes  (0.954103) | 6 | **-** | Cytoplasmic Membrane |

Letters in bold indicate proteome fraction where the protein has been identified. **SP** = surface protein, **CE** = cell envelope protein, **CMM** = protein over expressed on CMM, **Cyt** = cytoplasmic, **Ext** = extracellular, **Mem** = membrane.

**(1)** Spot numbers refer to Fig. 1, 2, & 3

**(2)** Scans a sequence against PROSITE or a pattern against the UniProt Knowledgebase (Swiss-Prot and TrEMBL)

**(3)** Presence of signal peptide predicted by SignalP 3.0.

**(4)** Non-classical i.e. non-signal peptide triggered protein secretion by SecretomeP. For each input sequence the server predicts the possibility of non-classical secretion. For bacteria, four scores are generated by the SecretomeP server for each input sequence. The determining score is the 'SecP score', for which a value above 0.5 indicates possible secretion. Values in parenthesis are SecP scores.

**(5)** Number of predicted transmembrane helices in proteins using TMHMM Server v. 2.0.

**(6)** Prediction of lipoproteins and signal peptides using LipoP 1.0**.**

**(7)** Prediction of protein subcellular localization using PSORT version 2.0.
